# The Potential Contribution of Hexavalent Chromium to the Carcinogenicity of Chrysotile Asbestos

**DOI:** 10.1021/acs.chemrestox.2c00314

**Published:** 2022-11-21

**Authors:** Martin Walter, Walter D.C. Schenkeveld, Maura Tomatis, Karin Schelch, Barbara Peter-Vörösmarty, Gerald Geroldinger, Lars Gille, Maria C. Bruzzoniti, Francesco Turci, Stephan M. Kraemer, Michael Grusch

**Affiliations:** † Department of Environmental Geosciences, University of Vienna, Althanstraße 14 (UZA II), 1090Vienna, Austria; ‡ Department of Veterinary Sciences, University of Torino, L.go Paolo Braccini, 2, Grugliasco, 10095 (TO), Italy; § “G.Scansetti” Interdepartmental Center for Studies of Asbestos and Other Toxic Particulates, Via Pietro Giuria, 7, 10125Torino, Italy; ∥ Center for Cancer Research, Medical University of Vienna, Borschkegasse 8a, 1090Vienna, Austria; ⊥ Institute of Pharmacology and Toxicology, University of Veterinary Medicine, Vienna, Veterinärplatz 1, 1210Vienna, Austria; # Department of Chemistry, University of Torino, Via Pietro Giuria, 7, 10125Torino, Italy

## Abstract

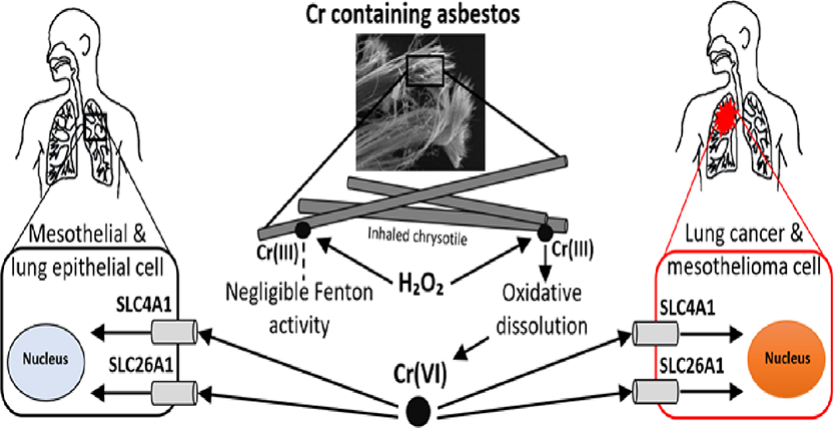

Chrysotile asbestos is a carcinogenic mineral that has abundantly been used in industrial and consumer applications. The carcinogenicity of the fibers is partly governed by reactive Fe surface sites that catalyze the generation of highly toxic hydroxyl radicals (HO^•^) from extracellular hydrogen peroxide (H_2_O_2_). Chrysotile also contains Cr, typically in the low mass permille range. In this study, we examined the leaching of Cr from fibers at the physiological lung pH of 7.4 in the presence and absence of H_2_O_2_. Furthermore, we investigated the potential of cells from typical asbestos-burdened tissues and cancers to take up Cr leached from chrysotile in PCR expression, immunoblot, and cellular Cr uptake experiments. Finally, the contribution of Cr to fiber-mediated H_2_O_2_ decomposition and HO^•^ generation was studied. Chromium readily dissolved from chrysotile fibers in its genotoxic and carcinogenic hexavalent redox state upon oxidation by H_2_O_2_. Lung epithelial, mesothelial, lung carcinoma, and mesothelioma cells expressed membrane-bound Cr(VI) transporters and accumulated Cr up to 10-fold relative to the Cr(VI) concentration in the spiked medium. Conversely, anion transporter inhibitors decreased cellular Cr(VI) uptake up to 45-fold. Finally, chromium associated with chrysotile neither decomposed H_2_O_2_ nor contributed to fiber-mediated HO^•^ generation. Altogether, our results support the hypothesis that Cr may leach from inhaled chrysotile in its hexavalent state and subsequently accumulate in cells of typically asbestos-burdened tissues, which could contribute to the carcinogenicity of chrysotile fibers. However, unlike Fe, Cr did not significantly contribute to the adverse radical production of chrysotile.

## Introduction

Asbestos is a term referring to a group of fibrous silicate minerals, which can be divided into amphibole and serpentine asbestos.^1,2^ The only member of the serpentine group is chrysotile asbestos, which accounts for more than 95% of the historical asbestos usage.^1−3^ Asbestos fibers have been heavily used in a variety of industrial, technical, and consumer applications, especially throughout the 20th century.^2,4^ Exposure to the fibers can induce malignant and nonmalignant diseases. According to the latest WHO-IARC monograph on asbestos, there is sufficient evidence that respiratory exposure to asbestos causes cancer of the lung, larynx, and ovaries and mesothelioma in the pleura and peritoneum.^5^ Nonmalignant diseases caused by asbestos include pneumoconiosis, pleural thickening, plaques, and effusions.^6^ In 2014, the WHO-IPCS estimated that worldwide, at least 107,000 people die annually as a result of asbestos exposure.^7^ Because of the toxicity and carcinogenicity of the fibers, the application of asbestos has been banned in many European countries starting from the late 1980s onward.^8−10^ In contrast to Europe, the usage of asbestos has not been banned yet in northern American countries like the United States and Mexico,^10^ and in Asian countries, it even increases.^11,12^

Since the historical use of asbestos is dominated by chrysotile,^3^ and the recent use is limited to this mineral,^2^ we have focused on chrysotile asbestos in this study. Chrysotile [Mg_3_Si_2_O_5_(OH)_4_] consists of octahedral Mg and tetrahedral Si layers, which bundle together to a fiber with an external Mg hydroxide surface.^13,14^ Dissolution of chrysotile at circumneutral pH is commonly described as a step-by-step dissolution of alternating Mg and Si layers.^15,16^ In chrysotile suspensions at pH 7.4, the outermost Mg layer dissolved within hours to days, rendering an exposed Si layer, which controlled further dissolution because of its slow dissolution kinetics.^15,17,18^ Also, in vivo, the outermost Mg layer of chrysotile rapidly dissolved within days after intrapleural administration in rats.^19^

The carcinogenicity of asbestos is largely determined by three fiber properties: a high biopersistence, a large aspect ratio, and a high chemical reactivity of the fiber surfaces resulting from reactive surface species.^9,20,21^ A well-examined mode of the chemical reactivity of chrysotile fibers is the redox cycling of Fe surface sites in the presence of (extracellular) H_2_O_2_, yielding highly toxic hydroxyl radicals (HO^•^).^20−23^ In this Haber–Weiss redox cycle, surface Fe^3+^ is reduced to Fe^2+^ by physiological reductants or H_2_O_2_ decomposition products such as hydroperoxyl radicals or superoxide. Fe^2+^, in turn, is back-oxidized by H_2_O_2_ in the Fenton reaction to yield Fe^3+^ and HO^•^.^21,22,24−29^ Recently, tetrahedrally coordinated Fe (Fe^3+^_tet_) was identified as the dominant Fenton-reactive Fe species on chrysotile surfaces after fiber weathering in the physiologically and environmentally relevant pH range (pH 3–9).^15,30^ The current study, however, focuses on the potential contribution from other transition metals, particularly Cr, to the chemical reactivity of chrysotile fibers. Apart from Fe, first order transition metals in chrysotile include Cr, Ni, Mn, Co, and Zn. Particularly, the Cr and Ni bulk contents of chrysotile (both in the low g kg^–1^ range) are highly enriched, relative to the common amphibole asbestos minerals amosite and crocidolite.^31−35^ In chrysotile, these trace metals substitute Mg, but they are also found in associated phase impurities in the raw chrysotile material.^33,36^ The content of both Cr and Ni also increases during the industrial processing of crude fibers, e.g., during crushing and milling with equipment from steel alloys that contain these metals.^37,38^ The concentrations of most trace metals can be larger in impurities from associated minerals and alloys than in chrysotile itself.^36^ In vivo, chromium and cobalt readily dissolved from intrapleurally administered chrysotile in rats, and both metals leached at much higher rates than Fe.^19,39^

Cr in serpentinite minerals (including chrysotile) is exclusively present in the trivalent redox state,^40,41^ which is poorly soluble at the physiological lung pH of 7.4.^42,43^ The oxidation state of Cr strongly determines its toxicokinetics and hence its genotoxicity and carcinogenicity.^44^ Cr(III) is more reactive toward nucleic acids than Cr(VI) but has a weak membrane permeability and is therefore unable to cross cell membranes.^45−48^ However, Cr(VI) predominantly occurs as the chromate oxyanion at circumneutral pH, which easily enters cells via anion transport carrier systems like the chloride/bicarbonate exchanger (SLC4A1) and the sulfate anion exchanger (SLC26A1).^46,49−51^ Inside the cell, Cr(VI) is subsequently reduced by common intracellular reductants.^52^ The reduced Cr(V), Cr(IV), or Cr(III) then exerts its intracellular genotoxic potential by inducing the formation of DNA adducts, DNA-strand-breaks, DNA-protein cross-links, oxidized bases, abasic sites, and DNA inter- and intrastrand cross-links.^44,45,51−55^ Apart from its direct genotoxicity, chromium can also undergo Fenton-like redox reactions and, therefore, exert oxidative stress to exposed cells and thus indirect genotoxicity.^56^ Owing to its high genotoxic and carcinogenic potential, the WHO-IARC classified Cr(VI) compounds as carcinogenic to humans (group 1); the panel concluded that there is sufficient evidence that Cr(VI) compounds cause cancer in the lung.^51,57^ Chrysotile also contains Ni, typically in a mass fraction comparable to Cr.^31^ Similarly to Cr, Ni has been classified as carcinogenic to humans (group 1) in the latest WHO-IARC monograph.^58^ However, contrary to Cr, Ni is a weak genotoxicant, and Ni-induced carcinogenicity is only governed by dose-dependent mechanisms, e.g., depletion of antioxidants and disruption of DNA repair mechanisms.^59−61^ Because of the aforementioned properties, in this study, we focused on the potential contribution of Cr to the carcinogenicity of chrysotile asbestos.

Even though the contribution of Cr to the carcinogenicity of chrysotile has been postulated before,^38,62−64^ the fate and reactivity of chrysotile-associated Cr in the lungs and pleura are still poorly understood. A fast release of Cr from chrysotile administered intrapleurally was observed in rats,^19,39^ which appears counterintuitive, as the solubility of Cr(III) (as contained in chrysotile) at the physiologic pH of 7.4 is low.^42,43^ Therefore, the leaching of Cr from chrysotile at this pH required investigation. Furthermore, the potential of cells from asbestos-exposed tissues and cancers to take up Cr from chrysotile has, to our knowledge, not been assessed. Finally, the potential contribution of Cr to HO^•^ generation by asbestos is also still unknown.

This study aimed to address these knowledge gaps. We hypothesized that Cr leaching from chrysotile at the physiological lung pH of 7.4 is enhanced by the oxidation of poorly soluble Cr(III) by H_2_O_2_, which is observed extracellularly at elevated concentrations in inflamed tissues, to the well-soluble, genotoxic, and carcinogenic Cr(VI). This hypothesis is based on studies which demonstrated that at circumneutral pH, H_2_O_2_ oxidizes Cr(III) to Cr(VI)^42,65^ at extracellular H_2_O_2_ concentrations that can be expected under (patho)physiologic conditions.^23,42^ We further hypothesized that Cr(VI) transporting proteins are ubiquitously expressed in cells of asbestos-exposed tissues and cancers and that these cells may consequently take up Cr(VI) into intracellular compartments in which Cr exerts its genotoxicity and carcinogenicity. Finally, we hypothesized that Cr associated with chrysotile fibers decomposes H_2_O_2_, participates in Haber-Weiss cycling and thus contributes to fiber-mediated HO^•^ generation.

These hypotheses were tested in batch experiments examining metal leaching, H_2_O_2_ decomposition, and HO^•^ generation using pristine and weathered chrysotile asbestos fibers and in PCR, immunoblot, and cellular Cr(VI) uptake experiments using cells of typical asbestos-burdened tissues and asbestos-induced cancers. The results from this study contribute to an improved understanding of the carcinogenicity of chrysotile asbestos.

## Experimental Procedures

### Materials and Fiber Characterization

All chemical reagents were purchased from VWR (unless specified otherwise) and were at least p.a. grade. Chrysotile was obtained from a commercial supplier in China (Shijiazhuang Mining IMP&EXP Trade Co). The Shijiazhuang chrysotile asbestos has been extensively characterized, as reported in Walter et al.^15^ Its Mg, Si, and Mn content was determined by fusion digestion, its Zn and Co content by neutron activation analysis (NAA), and its Fe, Ni, and Cr content by both fusion digestion and NAA, as reported in Walter et al.^15,66^ In Table 1, the molar Mg/Si ratio of Shijiazhuang chrysotile is close to the stoichiometric value of 1.5. The two major metal substituents in the fibers were Fe (∼20 g kg^–1^) and Al (7.4 g kg^–1^). Almost all Fe in the fibers is substituted into the octahedral Mg layer (∼93%), whereas only 7% constituted Fe^3+^_tet_. Minor substituted transition metals in Shijiazhuang chrysotile asbestos included Cr, Ni, and Mn (all ±1 g kg^–1^) and to a lesser extent, Co (∼54 mg kg^–1^) and Zn (∼17 mg kg^–1^).

**Table 1:** Bulk Properties of Shijiazhuang Chrysotile Asbestos, as Reported by Walter et al.^15,66^^,^^c^

bulk analyses of Shijiazhuang chrysotile asbestos			
**bulk metal and Si content:**		fusion digestion	NAA
Mg	[g kg^–1^]	253 (8.9)	
Si	[g kg^–1^]	193 (6.4)	
Fe	[g kg^–1^]	19.0 (1.2)	21.4 (0.3)^a^
Al	[g kg^–1^]	7.4 (0.8)	
Mn	[g kg^–1^]	0.8 (0.1)	
Ni	[g kg^–1^]	1.3 (0.10)	1.2 (0.03)
Cr	[g kg^–1^]	1.4 (0.11)	1.3 (0.04)
Zn	[mg kg^–1^]		17.0 (1.1)
Co	[mg kg^–1^]		53.6 (1.0)
**bulk Fe speciation:**		Mössbauer analysis	
Fe^2+^_oct_	[%]	38.4^a^	
Fe^3+^_oct_	[%]	54.6^a^	
Fe^3+^_tet_	[%]	7.0^a^	
total Fe in chrysotile^b^	[%]	68.2^a^	

a Taken from Walter et al. (2019),^15^ all other data taken from Walter et al. (2022).^66^

b The remaining Fe is in phase impurities including magnetite.

c Bulk metal and Si contents were determined by fusion digestions (*n* = 32) and by neutron activation analysis (NAA, *n* = 2), and the Fe bulk speciation was determined by Mössbauer ^57^Fe spectroscopy. Values in round brackets indicate standard deviations.

The Shijiazhuang chrysotile material also includes mineral impurities like brucite (4.5 ± 2.1%), talc (3.4 ± 2.0%), chlorite (2.4 ± 2.9%), and magnetite (1.5 ± 0.2%)^15^ that can also contain Cr. However, additional characterization complemented with the experimental results from this study suggests that Cr predominantly resides in and is mobilized from the chrysotile mineral. Details are provided in the Supporting Information (Figure S1 and associated text).

### Experimental Strategy

In this study, we explored two potential mechanisms through which Cr associated with chrysotile asbestos may contribute to chrysotile’s carcinogenicity: (1) through oxidative mobilization of Cr(III) from chrysotile by H_2_O_2_ and subsequent uptake of Cr(VI) into lung epithelial, mesothelial, lung carcinoma, and mesothelioma cells and (2) through the generation of reactive oxygen species by Cr on chrysotile surfaces.

Regarding the first mechanism, initially, the size of the Cr pool available for mobilization from chrysotile by H_2_O_2_ at the physiological lung pH of 7.4 was assessed by incubating pristine fibers in solutions containing chelators with a high affinity for Cr(III). Subsequently, the potential of H_2_O_2_ to promote Cr leaching from pristine and weathered (preconditioned) chrysotile was examined in batch experiments. Finally, the ability of lung epithelial, mesothelial, lung carcinoma, and mesothelioma cells to take up (mobilized) Cr was assessed in PCR, immunoblot, and cellular Cr(VI) uptake experiments.

Regarding the second mechanism, the potential contribution of Cr associated with chrysotile asbestos to Haber–Weiss cycling was examined in batch experiments assessing H_2_O_2_ decomposition and HO^•^ generation by pristine chrysotile and preconditioned chrysotile with either a high or a low surface Cr content.

### Metal Leaching, H_2_O_2_ Decomposition, and HO^•^ Generation

#### Fiber Preconditioning

Prior to the experiments, pristine Shijiazhuang chrysotile asbestos was preconditioned to obtain weathered chrysotile fibers with specific surface properties. Preconditioning was conducted according to Walter et al.;^30^ the fibers were incubated for 336 h in blank or ligand (1 mmol L^–1^ of the siderophore desferrioxamine-B (DFOB; Novartis)) solutions buffered at pH 7.4 to obtain “blank-altered fibers” or “DFOB-altered fibers”, respectively. During preconditioning, the outermost Mg layer of pristine fibers is dissolved, and its Fe content either precipitates as low Fenton-active secondary Fe phases (blank-altered fibers) or becomes complexed and mobilized by DFOB (DFOB-altered fibers).^15^ Preconditioning with DFOB additionally removes the Fe^3+^_tet_ content from the outermost Si layer.^15,30^ In the context of this study, blank-altered fibers represent chrysotile that has weathered in the absence of metal chelators (e.g., in occupational and household settings), and DFOB-altered fibers represent chrysotile that had weathered in the presence of naturally occurring metal chelators (e.g., biotic ligands^15,67−69^). Analogously to preconditioning with DFOB, two additional fiber types were prepared by incubating pristine chrysotile fibers for 336 h in the presence of the synthetic chelating ligands diethylenetriaminepentaacetic acid (DTPA; Sigma Aldrich) and ethylenediaminetetraacetic acid (EDTA; Sigma Aldrich) (“DTPA-altered fibers” and “EDTA-altered fibers”, respectively). DTPA and EDTA have a high affinity for Cr(III)^70,71^ and were used to effectively deplete chrysotile surfaces of Cr, e.g., for determining H_2_O_2_ decomposition and HO^•^ generation by fibers with a low surface Cr content.

#### General Experimental Procedure

Experiments and preconditioning of chrysotile were carried out in fiber suspensions with a solid-to-solution ratio of 1 g L^–1^ (unless mentioned otherwise). The nonmetal-complexing tertiary amine (“Better”) buffer^72^ MOPS (3-(*N*-morpholino)propane sulfonic acid) was used at a concentration of 50 mmol L^–1^ to buffer fiber suspensions at the physiological lung pH of 7.4; the pH was maintained throughout the experiments within mainly ±0.3 pH units. For selected treatments, Cr dissolution from chrysotile was studied at pH 3.0 ± 0.3, using 50 mmol L^–1^ of the (“Better”) buffer^72^ PIPPS (1,4-piperazinedipropanesulfonic acid) to fix the pH. The ionic strength (IS) of the suspensions was adjusted to 300 mmol L^–1^ by addition of NaCl to facilitate comparisons with previous studies on Shijiazhuang chrysotile at the same IS.^15,30^ Experiments were either carried out in the absence of ligands (buffer-only “blank” treatments) or in the presence of 1 mmol L^–1^ of the synthetic metal chelators DTPA, EDTA, or the bacterial siderophore DFOB. DTPA and EDTA were selected for their high affinity for Cr(III).^70,71^ DFOB was selected because it has been extensively used in metal leaching studies with asbestos fibers^15,22,30,73,74^ and was shown to dissolve Cr from hydroxide minerals in a nonoxidative fashion.^75^ Metal leaching and H_2_O_2_ decomposition experiments were carried out in duplicates. Suspensions were prepared in 15 mL PP-tubes (VWR or Greiner) and were incubated in an end-over-end shaker rotating at 15 rounds per minute (RPM) at 20 ± 2 °C in the dark (unless specified otherwise). For experiments involving H_2_O_2_, by default, a 30% stock solution (Sigma Aldrich, trace analysis) was diluted 100× to obtain an initial experimental H_2_O_2_ concentration of 3.3 g L^–1^ (∼0.3%, i.e., ∼100 mmol L^–1^). This concentration is substantially larger than H_2_O_2_ concentrations observed in inflamed lung tissue. However, an additional leaching experiment was conducted at pH 7.4 in the presence of 100, 10, 1, and 0.1 mmol L^–1^ H_2_O_2_; the latter two levels represent extracellular H_2_O_2_ concentrations in inflamed tissues.^23^ Samples in this H_2_O_2_ dilution experiment contained a 10-fold lower NaCl background concentration (suprapure quality, Sigma) at 25 mmol L^–1^ to facilitate ultra-trace metal analysis and were shaken in an orbital shaker at 20 RPM. The H_2_O_2_ concentration of the stock was determined by redox titration with KMnO_4_ prior to the experiments and amounted 334 ± 2 g L^–1^ H_2_O_2_. Cr speciation of selected samples from the leaching experiments were analyzed by an LC-ICP-MS method (vide infra). For this purpose, 1 g L^–1^ suspensions of chrysotile were incubated in an orbital shaker at 20 RPM at pH 3.0 and 7.4 at an IS of 300 mmol L^–1^. Cr speciation was additionally analyzed in variations of these two treatments: at 10-fold decreased NaCl background at pH 7.4 and in the absence of an organic buffer. Fibers were incubated for up to 336 h, and the suspensions were sampled destructively after 0.5, 1, 2, 4, 8, 24, 48, 96, 168, and 336 h. For solution sampling, fiber suspensions were filtered over a 0.45 μm Sartorius cellulose acetate syringe filter (VWR). An aliquot of the filtrate was acidified with trace metal grade HNO_3_ to 0.14 mol L^–1^ and kept in a refrigerator until analysis. In experiments involving H_2_O_2_, another aliquot was immediately analyzed for its H_2_O_2_ concentration. Samples for Cr speciation analyses were kept at room temperature until LC-ICP-MS analysis. Fibers were sampled by vacuum filtration using a 0.45 μm Nylon membrane (Magna) in a Büchner funnel. Subsequently, the fibers were washed with ultra-pure water to remove potentially adsorbed free ligands or metal complexes. Finally, the fibers were vacuum-dried and kept in an evacuated desiccator for follow-up experiments or EPR analyses.

#### Analytical Procedures

Total dissolved Cr, Ni, Fe, Mn, Zn, Co, Mg, Al, and Si concentrations in the acidified sample filtrates were analyzed by ICP-OES (Optima 5300-DV, Perking Elmer) or by ICP-MS (Agilent 7700). The calibration standards were matrix-matched with the samples.

Cr speciation in leachates was determined by LC-ICP-MS (a chromatographic column for Cr speciation (G3268-80001, Agilent) was coupled to ICP-MS (Agilent 7700)). Before analysis, the pH of aliquots of fiber leachates was adjusted to pH 7, EDTA was added to a concentration of 50 mmol L^–1^, and samples were heated to 60 °C for 1 h. An isocratic pump (Postnova analytics) was then used to pump the mobile phase (a 50 mmol L^–1^ EDTA solution at pH 7) through the column at a flow rate of 1.5 mL/min and an injection volume of 100 μL. Cr(III)-EDTA complexes and Cr(VI) were separated in the column as Cr(VI) had a longer retention time (*t*_R_ > 5 min) than Cr(III)-EDTA complexes (*t*_R_ < 4 min). Finally, the Cr concentration was measured by ICP-MS (detection of ^52^Cr) at an RF power of 1550 W in He-mode (He being the carrier gas).

H_2_O_2_ concentrations in the filtrates were analyzed spectrophotometrically immediately after sampling with a Varian Cary 50 UV/VIS spectrophotometer according to a procedure reported in Walter et al.^30^ Control samples to determine H_2_O_2_ decomposition in the absence of fibers were also included as H_2_O_2_ reacts with the MOPS buffer.^76^

The fiber-mediated HO^•^ generation in the presence of H_2_O_2_ was determined using an X-band EPR spectrometer (Bruker EMX) and a split ring resonator (Bruker MD5). DMPO (5-5′-dimethyl-1-pyrroline-*N*-oxide) was used as spin-trapping agent. This technique has frequently been applied before to determine the HO^•^ generation by asbestos fibers,^15,24,25,28,68,77^ and was carried out according to Walter et al.^15^ EPR measurements were done in quadruplicates. The signal intensity (Ipp, intensity peak-to-peak) provides a measure for the HO^•^ yield of the fibers. The Ipp of altered fibers was expressed as a percentage of the Ipp of pristine fibers (defined as 100%), which was measured as a reference in each measurement session. Intensities of DMPO/HO^•^ spectra were quantified by the height of the second peak from the left in the quadruplet.

### Expression of Cr(VI) Anion Transporters and Cellular Cr(VI) Uptake

#### Cell Culture

For immunoblots and Cr(VI) uptake experiments, cultures of human immortalized lung epithelial (BEAS-2B), immortalized mesothelial (MeT-5A), lung carcinoma (A549), and malignant pleural mesothelioma (P31) cells were used. For PCR analysis, a panel containing two additional primary mesothelial (NP1, NP2) and four additional mesothelioma (MM05, SPC212, VMC23, VMC40) cell cultures was used. Gene expression microarray data were generated from MeT-5A, NP2, and a total of *n* = 35 mesothelioma cell cultures. Cells were maintained in growth medium supplemented with 2 g L^–1^ NaHCO_3_ and 10% heat-inactivated fetal bovine serum (both Thermo Fisher Scientific) in a humidified atmosphere (5% CO_2_, 37 °C). Establishment of malignant pleural mesothelioma cell cultures from surgical samples and cell line authentication were performed as published before.^78^ Cells were routinely monitored for mycoplasma contamination. Cell type, source, and culture medium for all cell cultures are listed in Table S1.

#### PCR Analysis

Cells were grown to 60–80% confluence before RNA was isolated using the innuPREP RNA Mini Kit (Analytik Jena AG) according to the manufacturer’s instructions and reverse transcribed with M-MLV reverse transcriptase (Thermo Fisher Scientific). The expression of the anion transporter solute carrier family 4, anion exchanger, member 1 (SLC4A1, a chloride/bicarbonate exchanger) and solute carrier family 26, anion exchanger, member 1 (SLC26A1, a sulfate/anion exchanger) were analyzed by qPCR (iTaq Universal SYBR Green Supermix, BioRad) on a CFX96 Touch thermocycler (BioRad) using the following primers: SLC4A1_for: 5′GCAACAGCCACAGACTAC; SLC4A1_rev: 5′TGCAGCTCCACATAGACC; SLC26A1v1-3_for: 5′GGCCATCGCCTACTCATTG; SLC26A1v1-3_rev: 5′GAGGTTGGCGAAGAAGGAC; GAPDH_for: 5′AGCTCACTGGCATGGCCTTC; GAPDH_rev: 5′ACGCCTGCTTCACCACCTTC; β-actin_for: 5′ACTCTTCCAGCCTTCCTTC; β-actin_rev: 5′GATGTCCACGTCACACTTC. The amplification efficiencies were 96.5, 96.3, 102.5 and 104.3% for SLC4A1, SLC26A1, GAPDH and β-actin, respectively (Figure S2). Gene expression levels are shown as 2^–Δ*Ct*^ × 10^6^ values normalized to both beta-actin and GAPDH as reference genes.

#### Gene Expression Microarray

Total RNA was isolated using TRIZOL (Thermo Fisher Scientific), and quantity and integrity (RIN > 8) were determined using an Agilent 2100 Bioanalyzer (Agilent Technologies). Genome-wide transcriptomic analysis was carried out using 4 × 44K whole genome gene expression arrays as described.^79^ Labeling and hybridization processing were performed as per the manufacturer’s instructions. Arrays were scanned on an Agilent G2505B microarray scanner and analyzed using GeneSpring version 13.0.4 GX.

#### Immunoblots

Cells were lysed in protein lysis buffer (150 mM NaCl, 50 mM Tris, 1 mM EGTA, 1 mM Na_3_VO_4_, 10 mM NaF, 1% Triton X-100 and 1× proteinase inhibitor mix from Roche), and protein concentration was determined with a Bradford protein assay from BioRad following the manufacturer′s instructions. For analysis of SLC26A1 and SLC41A, 30 and 80 μg, respectively, of total protein per lane were separated by SDS polyacrylamide gel electrophoresis and subsequently electroblotted onto PVDF membranes. Membranes were blocked in 5% milk powder for 1 h and subsequently incubated with the following primary antibodies overnight at 4 °C: rabbit polyclonal anti-SLC26A1/SAT1, 1:200, Proteintech, rabbit monoclonal anti-SLC4A1, 1:100, Cell Signaling Technology and mouse monoclonal anti-β-actin, 1:3000, Sigma. After washing with Tris-buffered saline with 0.1% Tween 20, membranes were incubated in horseradish peroxidase-coupled secondary antibodies (Agilent) for 1 h at room temperature. Luminescence signals were developed with Clarity Western ECL substrate (BioRad) and recorded on X-ray film.

#### Cellular Uptake of Cr(VI)

Cells (1 × 10^6^) were seeded into 10 cm petri dishes in medium with 10% heat-inactivated fetal bovine serum and grown for 4 days to reach a confluence of approximately 80–90%. Then, the medium was replaced with a serum-free medium and the anion transport inhibitor 4,4′-diisothiocyanostilbene-2,2′-disulfonic acid (DIDS, Merck) and niflumic acid (NA, Merck) were added at a concentration of 200 μmol L^–1^ where indicated. Inhibitor concentrations were chosen according to previous literature reports.^80,81^ After 1 h, Na_2_CrO_4_ (Merck) was added at final concentrations ranging from 0.2 to 2000 μmol L^–1^. After an incubation period of 4 h, the medium was removed, and cells were briefly washed with cold PBS and subsequently scraped into 1.5 mL tubes. Samples were snap frozen in dry ice and stored at −80 °C. On the next day, samples were subjected to three freeze–thaw cycles and sonicated for 30 min to break up the cells. Insoluble material was pelleted by centrifugation, and 40 μL of supernatant was pipetted into 500 μL of 10% trace metal grade HNO_3_ and stored at 4 °C. One μL of each sample was used to analyze protein concentration with a Bradford protein assay kit. The acidified 40 μL of the obtained supernatants were diluted with ultrapure water and subsequently filtered over a 0.45 μm Sartorius cellulose acetate syringe filter (VWR). The Cr concentration in the filtrate was then analyzed by ICP-MS (Agilent 7700 & 7900).

#### Cytotoxicity Test

Cells were seeded as described above for the cellular uptake experiment and treated with the highest concentrations of DIDS, niflumic acid, and Na_2_CrO_4_ used in the cellular uptake experiment. For the last hour of the experiment, the fluorescent dyes Hoechst 33258 and propidium iodide (both from Merck) were added at concentrations of 500 and 200 μmol L^–1^, respectively. Cytotoxicity, indicated by cellular uptake of propidium iodide, was monitored under a Nikon Ti 300 fluorescence microscope 1 h later.

### Statistical Analyses

Statistical analyses were performed with Graph Pad Prism 8 (where indicated). One-way ANOVA with Sidak′s multiple comparison test was used for comparison of multiple groups. A *p*-value of <0.05 was considered significant.

## Results

### Leaching of Cr from Chrysotile

**Figure 1:**
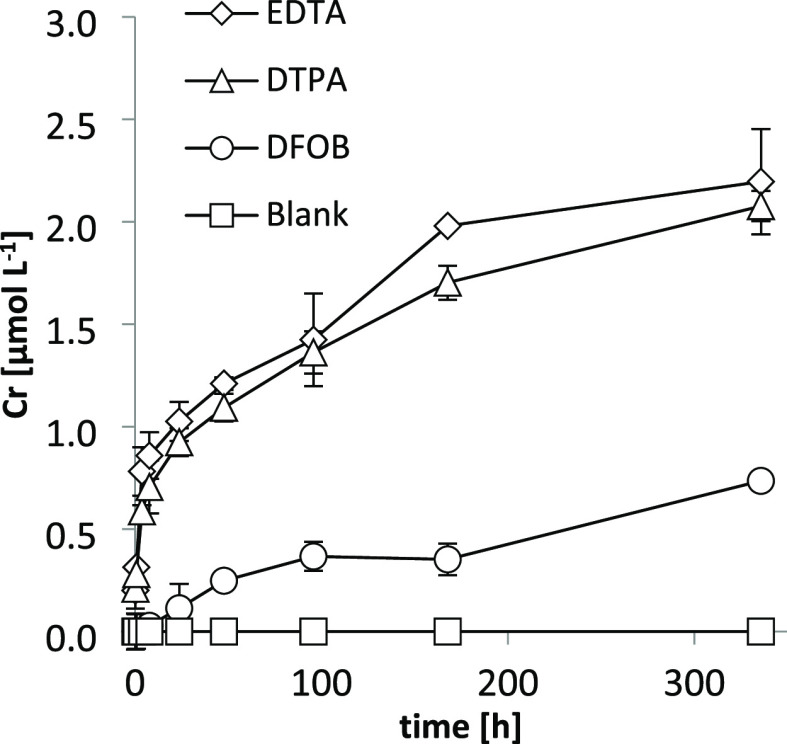
Cr concentrations (in μmol L^–1^) mobilized from 1 g L^–1^ pristine fibers at pH 7.4 by 1 mmol L^–1^ of the synthetic chelators DTPA and EDTA and the siderophore DFOB and in the absence of ligands (blank). Error bars indicate standard deviations (*n* = 2). Data presented in this figure are reported in Table S2.

Mobilized Cr concentrations from pristine fibers by metal chelators were the largest in the EDTA and DTPA treatments (Figure 1, Table S2). For example, after 336 h, EDTA and DTPA had mobilized 2.2 and 2.1 μmol L^–1^ Cr, respectively. Chromium mobilization by DFOB was less efficient: only 0.7 μmol L^–1^ Cr had leached after 336 h. In the blank treatment, Cr leaching from fibers was below the limit of detection (LOD) (16.6 nmol L^–1^) throughout the incubation (Figure 1). EDTA and DTPA were also more efficient at mobilizing Fe and Ni from pristine fibers than DFOB, and leached concentrations in blank solutions were below LOD for Fe and in the lower submicromolar range for Ni (Figure S3).

**Figure 2:**
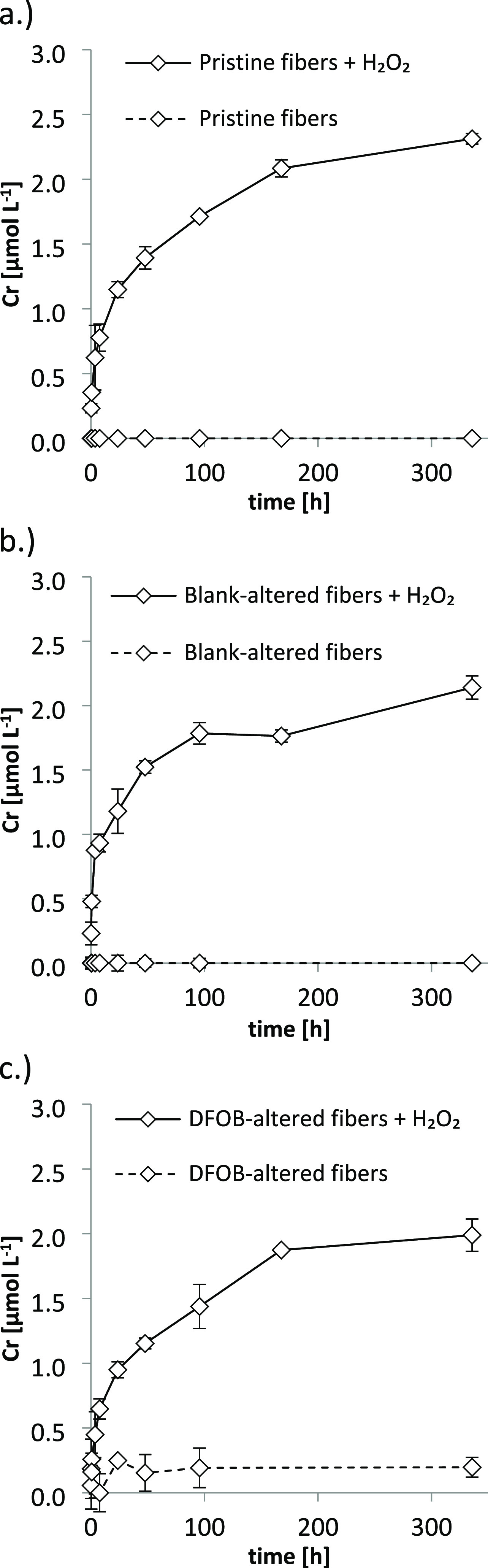
Cr concentrations (in μmol L^–1^) mobilized from 1 g L^–1^ pristine (panel a), blank-altered (panel b), and DFOB-altered chrysotile fibers (panel c) as a function of time at pH 7.4, either in the presence or absence of 3.3 g L^–1^ H_2_O_2_ (∼100 mmol L^–1^) (starting concentration). Error bars indicate standard deviations (*n* = 2). Data presented in this figure are reported in Table S3.

In experiments examining H_2_O_2_-mediated Cr leaching from pristine and weathered fibers (Figure 2, Table S3), mobilized Cr concentrations in the blank control treatments (no H_2_O_2_ added) also remained below the LOQ for pristine and blank-altered fibers (Figure 2a,b, respectively) and in the low submicromolar range for DFOB-altered fibers (Figure 2c). However, in the presence of H_2_O_2_, mobilized Cr concentrations were considerably elevated for all three fiber types. The Cr mobilization rate was initially large; the average rate for the three fiber types during the first 8 h was 0.93 pmol m^–2^ s^–1^ (calculated according to Walter et al.^15^), but the rate gradually declined. Cr mobilization was similar for the three fiber types and reached a concentration between 2.0 and 2.3 μmol L^–1^ after 336 h. H_2_O_2_ leached Cr from chrysotile to a similar extent as the ligands EDTA and DTPA (Figure 1).

Also, at lower applied H_2_O_2_ concentrations, Cr leaching from pristine chrysotile at pH 7.4 was observed. For 1 mmol L^–1^ H_2_O_2_, approximately 0.4 μmol L^–1^ Cr were still mobilized after 168 h (Figure S4), indicating that Cr leaching can occur at H_2_O_2_ concentrations down to the submillimolar range.

In the absence of MOPS buffer (leading to an increase in suspension pH to 8.9), Cr was also only mobilized from pristine chrysotile if H_2_O_2_ was added to the suspension. Mobilized Cr concentrations were smaller than observed in samples buffered at pH 7.4 due to the slower fiber dissolution kinetics at higher pH, as illustrated by the lower mobilized Mg concentrations (Table S4). At pH 3.0, however, Cr dissolution from pristine chrysotile fibers was clearly higher than in the presence of H_2_O_2_ at pH 7.4, reaching up to 6.1 μmol L^–1^ after 336 h (Figure S5).

For other heavy metals, like Ni, a pronounced effect of H_2_O_2_ on leaching at pH 7.4 was not observed, particularly with blank-altered and DFOB-altered fibers (Figure S6). Additionally, mobilization by DTPA, EDTA, and DFOB was considerably larger than by H_2_O_2_, as exemplified for Ni in Figures S3 and S6.

In order to test whether Cr leaching from chrysotile at pH 7.4 renders fiber surfaces with a lower surface Cr content, Cr leaching from ligand-pretreated fibers was examined in the presence of either H_2_O_2_ or DFOB under the same experimental conditions. During preconditioning of the fibers, 1.9, 2.2, and 0.7 μmol g^–1^ Cr had been removed by DTPA, EDTA, and DFOB, respectively (Table 2), corresponding with the results presented in Figure 1. In the subsequent leaching experiment, only marginally smaller amounts of Cr were leached from DTPA- and EDTA-altered fibers in the presence of H_2_O_2_ or from DFOB-altered fibers in the presence of DFOB (Table 2). Contrary to Cr, ligand preconditioning rendered fiber surfaces that were considerably depleted in Fe and Ni (Table S5). For example, DFOB removed on average ∼30 μmol g^–1^ Fe during preconditioning, whereas it only removed 4 μmol g^–1^ Fe during the subsequent incubation. Similarly to Fe, mobilized Ni concentrations by DFOB from DFOB-altered fibers (0.3 μmol g^–1^) were almost 6 times lower than the Ni concentrations that were mobilized from pristine fibers during the DFOB preconditioning of the fibers (1.7 μmol g^–1^).

**Table 2:** Removal of Cr from Fibers at pH 7.4 during Preconditioning with 1 mmol L^–1^ Ligand or Blank Solutions for 336 h (First Column) and during Interaction of the Pristine and Preconditioned Fibers with a 3.3 g L^–1^ H_2_O_2_ Solution (Second Column) or a 1 mmol L^–1^ DFOB Solution (Third Column) for 336 h^a^

	Cr removal during preconditioning with or without ligands	Cr removal from (preconditioned) fibers by 3.3 g L^–1^ H_2_O_2_	Cr removal from preconditioned fibers by 1 mmol L^–1^ DFOB
	[μmol g^–1^]	[μmol g^–1^]	[μmol g^–1^]
pristine fibers		2.3 (0.0)	
DFOB-altered fibers	0.7	2.0 (0.1)	0.5 (0.1)
DTPA-altered fibers	1.9 (0.1)	1.7 (0.1)	
EDTA-altered fibers	2.2 (0.2)	1.9 (0.1)	
blank-altered fibers	0	2.1 (0.1)	

a Values in round braces indicate standard deviations (*n* = 2). Standard deviations could not be determined for fiber preconditioning carried out in a single container.

### Redox Speciation of Dissolved Cr

**Figure 3:**
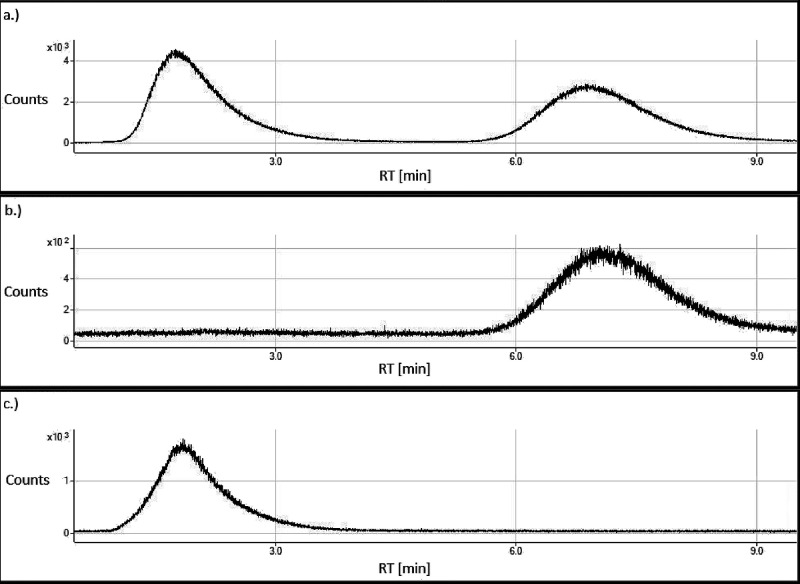
Cr redox speciation of selected samples from the leaching experiments (all at an ionic strength of 300 mmol L^–1^) determined by LC-ICP-MS. For the chromatographic conditions, see the experimental section. The Cr(III) retention time was <4 min, whereas the Cr(VI) retention time was >5 min. (a) Cr(III) (left peak) and Cr(VI) (right peak) standard, both at 50 ppb, used as upper limit for the calibration of the LC-ICP-MS speciation method. (b) Leachate collected after incubation of 1 g L^–1^ chrysotile at pH 7.4 for 168 h at an initial H_2_O_2_ concentration of 3.3 g L^–1^ (∼100 mmol L^–1^) (sample was diluted 10 times). (c) Leachate collected after incubation of 1 g L^–1^ chrysotile at pH 3.0 for 168 h (sample was diluted 20 times). The corresponding Cr redox species concentrations in the leachates for which the chromatograms are presented in panels (b) and (c) are reported in Table S4; full chromatograms up to approximately 15 min retention time are presented in Figure S7.

LC-ICP-MS analyses demonstrated that Cr leached from chrysotile in the presence of H_2_O_2_ at pH 7.4 was exclusively Cr(VI) (Figure 3, Table S4). Also, in the absence of MOPS buffer, Cr that leached from chrysotile asbestos in the presence of H_2_O_2_ was exclusively Cr(VI) (Table S4). In contrast, in pristine chrysotile fiber suspensions at pH 3.0 (to which no H_2_O_2_ was added), the leached Cr was exclusively Cr(III) (Figure 3, Table S4). The chromatograms of all LC-ICP-MS speciation analyses are presented in Figure S7. In addition to the LC-ICP-MS speciation analyses, the solution speciation of the experimental sample from the H_2_O_2_ treatment at pH 7.4 with the largest mobilized Cr concentration (Figure 2a) was predicted with the geochemical modeling program PHREEQC and the SIT database^82^ (Table S6). Equilibrium modeling suggested that practically all Cr was present in the hexavalent redox state, predominantly as CrO_4_^2–^ and to a smaller extent as HCrO_4_^–^. Only minor traces of Cr(III) (∼10^–15^ μmol L^–1^) were predicted.

### Expression of Cr(VI) Transporters in Cells of Typical Asbestos-Burdened Tissues

**Figure 4:**
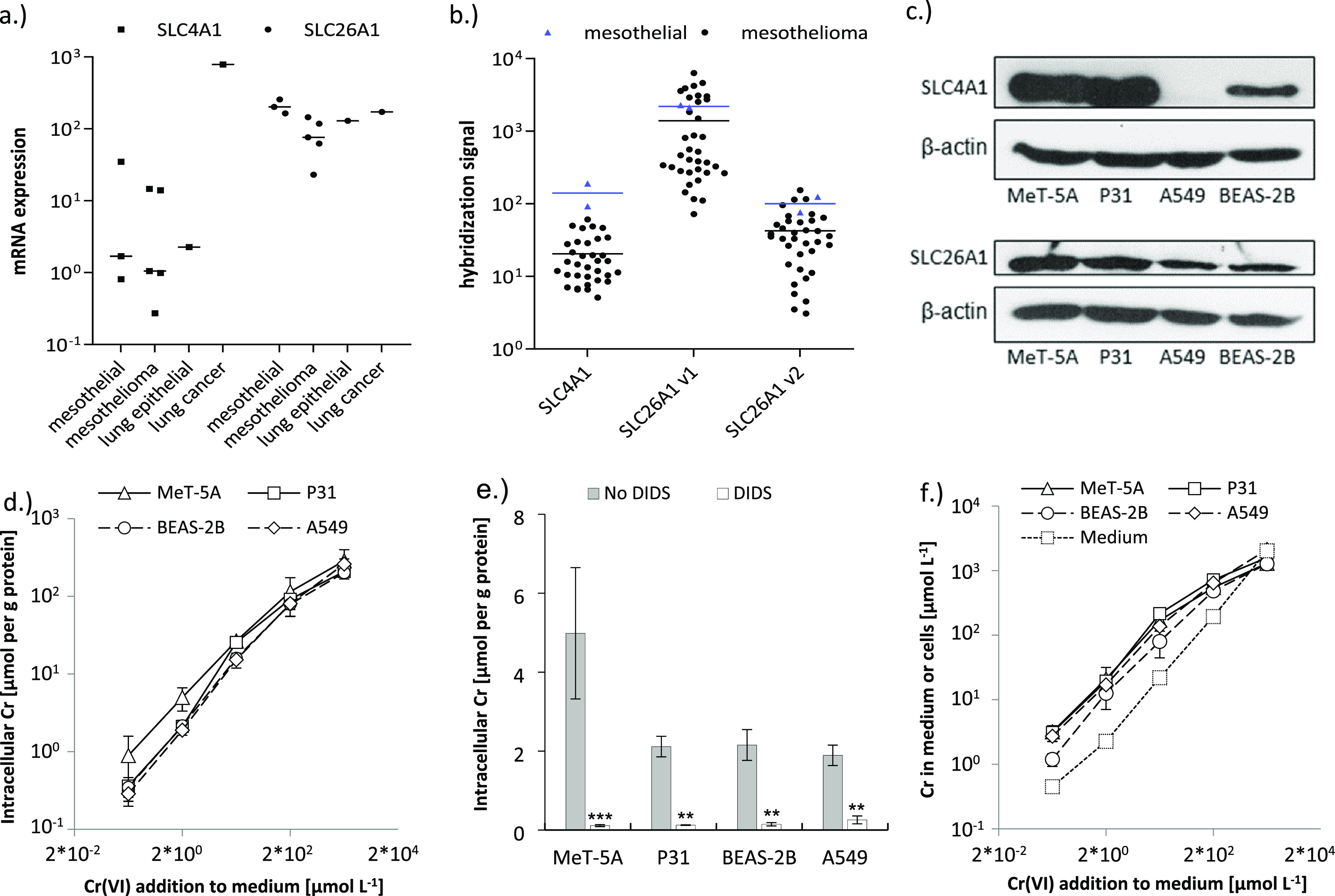
(a) Expression of SLC4A1 and SLC26A1 in mesothelial (MeT-5A, NP1, NP2), mesothelioma (P31, MM05, SPC212, VMC23, VMC40), lung epithelial (BEAS-2B), and lung carcinoma (A549) cells assessed by qPCR. All expression values were calculated as 2^–d*CT*^ × 10^6^ relative to two housekeeping genes (β-actin and GAPDH). Medians for each category (horizontal lines) and expression levels of individual cell lines are shown. (b) Expression levels of SLC4A1 and SLC26A1 (two oligos, v1 and v1) in a panel of mesothelial (*n* = 2) and mesothelioma (*n* = 35) cells extracted from Agilent 44 K microarray data, shown as raw hybridization signal. Horizontal lines indicate mean values of the presented data. (c) Immunoblots of the SLC4A1 and SLC26A1 proteins in MeT-5A, P31, BEAS-2B, and A549 cells. β-actin was used as loading control. (d) Cr(VI) uptake in MeT-5A, P31, BEAS-2B, and A549 cells when either 0.2, 2, 20, 200, and 2000 μmol L^–1^ Cr(VI) were spiked into the cell incubation media. Mean background protein normalized intracellular Cr concentrations ranged from 18 (P31) to 57 (MeT-5A) nmol g^–1^ (Table S7). (e) Cr(VI) uptake in MeT-5A, P31, BEAS-2B, and A549 cells in the presence and absence of 200 μmol L^–1^ of the anion transporter inhibitor DIDS when the cell incubation media had initially been spiked with 2 μmol L^–1^ of Cr(VI)). (f) Accumulation of Cr(VI) in the intracellular compartment of MeT-5A, P31, BEAS-2B, and A549 cells as compared to measured Cr(VI) concentrations in the cell media alone (”Medium” replicates) when 0.2, 2, 20, 200, and 200 μmol L^–1^ Cr(VI) were spiked into the cell media. Measured intracellular Cr contents [μmol L^–1^] in panels (d) and (e) were normalized to the cellular protein content [g L^–1^]. Data to this Figure are presented in Table S7 (the plotted data) and Table S1 (list of used cell lines, their type and source). **p < 0.005, ***p < 0.001 DIDS versus no DIDS. Error bars in panels (d) and (e) represent standard deviations (*n* = 3–5).

Expression of the Cr(VI) transporters SLC4A1 and SLC26A1 was tested in a panel of cultured cells (Table S1) derived from typical tissues exposed to asbestos and from asbestos-related cancers. Gene expression of both SLC4A1 and SLC26A1 was confirmed in lung epithelial, mesothelial, lung carcinoma, and mesothelioma cells by qPCR (Figure 4a). Furthermore, since especially mesothelioma is associated with asbestos exposure, expression arrays of a larger panel of mesothelioma cell lines (*n* = 33) were analyzed and SLC4A1 and SLC26A1 expression compared to nonmalignant mesothelial (*n* = 2) cells (Figure 4b). Consistent with our qPCR data, SLC26A1 generally showed higher expression than SLC4A1. Also, the nonmalignant mesothelial cells tended to express higher levels of both SLC4A1 and SLC26A1 than mesothelioma. To confirm the expression of SLC4A1 and SLC26A1 on the protein level, MeT-5A (mesothelial), P31 (mesothelioma), BEAS-2B (lung epithelial), and A549 (lung cancer) cells were further investigated by immunoblotting (Figure 4c). With the exception of A549, which showed only a faint band for SLC4A1, all cell types showed expression of both transporter proteins.

### Intracellular Cr Contents in Cellular Cr(VI) Uptake Experiments

The capability of the SLC4A1 and SLC26A1 protein expressing cells MeT-5A, P31, BEAS-2B, and A549 to take up Cr(VI) was tested by incubating them with medium containing 0.2 to 2000 μmol L^–1^ Na_2_CrO_4_ and measuring intracellular Cr by ICP-MS. The results show a concentration-dependent uptake of Cr(VI) that was comparable in lung-derived and mesothelium-derived cells (Figure 4d). Interestingly, at the highest tested Cr(VI) concentration (2000 μmol L^–1^), intracellular Cr contents were lower in all four cell types than would be extrapolated from the extent of intracellular Cr measured at lower spiked-in Cr(VI) levels. Incubating the cells with 200 μmol L^–1^ of the anion transporter inhibitor DIDS led to significantly reduced intracellular Cr concentrations in all cell models when 2 μmol L^–1^ of Cr(VI) had initially been spiked into the cell media, with the highest decreases in cellular Cr contents amounting to 45-fold in MeT-5A cells (Figure 4e). In the mesothelium-derived cells, a second inhibitor, niflumic acid (NA), was tested. It led to a significant decrease in intracellular Cr levels only in MeT-5A and showed no further reduction when used in combination with DIDS (Figure S8a). When 2000 μmol L^–1^ of Cr(VI) were spiked to the media, 200 μmol L^–1^ of DIDS caused a larger than equimolar decrease of intracellular Cr contents (Figure S8b). Incubation with Cr(VI) at the highest concentration with both inhibitors did not result in increased cytotoxicity compared to untreated cells (Figure S9). Finally, in cell-free medium, measured Cr concentrations were identical for replicates where no inhibitor was added and replicates in which 200 μmol L^–1^ of DIDS, NA, or DIDS + NA were added to the medium (Figure S10).

The intracellular Cr concentrations measured 4 h after incubation of the cells with Cr(VI) were up to 10 times higher than the Cr(VI) concentrations that had initially been spiked into the cell media (Figure 4f). This effect was highest in replicates of the three lowest applied Cr(VI) concentrations (0.2, 2, and 20 μmol L^–1^). At 200 μmol L^–1^ of spiked Cr(VI), accumulation of Cr in the intracellular compartment was already less, and at 2000 μmol L^–1^ of spiked Cr(VI), intracellular Cr contents in BEAS-2B, MeT-5A, and P31 cells were even below the initially spiked Cr(VI) concentrations in the cell media.

### H_2_O_2_ Degradation and HO^•^ Generation by Preconditioned Fibers

**Figure 5:**
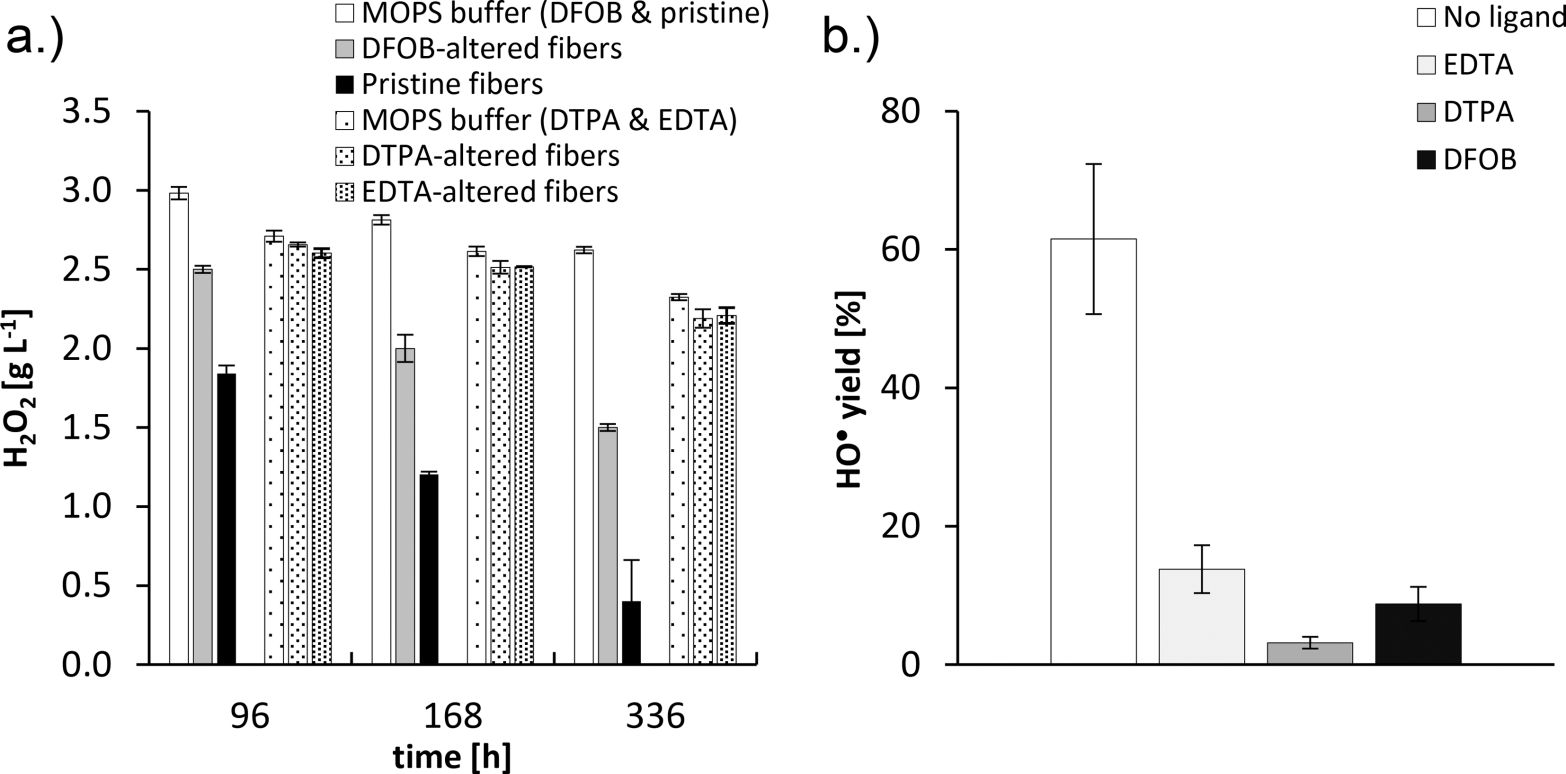
(a) H_2_O_2_ degradation at pH 7.4 in the absence of fibers (“MOPS buffer”), by pristine fibers and by fibers that had been preconditioned for 336 h at pH 7.4 in the presence of 1 mmol L^–1^ EDTA, DTPA or DFOB. The starting H_2_O_2_ concentration was 3.3 g L^–1^ (∼100 mmol L^–1^). The data were collected in two separate experiments; hence, for both experiments, the MOPS control treatment is reported (the MOPS (DTPA & EDTA) and MOPS (DFOB & pristine) columns). (b) Hydroxyl radical generation by fibers that were preconditioned in the absence of ligands or with DTPA, EDTA, and DFOB. A hydroxyl radical yield of 100% corresponds to the radical generation by pristine fibers (no preconditioning). Error bars indicate standard deviations (*n* = 2 in panel (a) and *n* = 4 in panel (b)). Data from the pristine fiber and DFOB treatment and the corresponding MOPS buffer control in panel (a) were taken from Walter et al.^30^ Data presented in this figure is reported in Table S8.

H_2_O_2_ decomposition was the fastest for pristine fibers, with only 0.4 g L^–1^ H_2_O_2_ (∼12%) remaining after 336 h (Figure 5a). Fibers from which transition metals had (partly) been leached from the surfaces during preconditioning with ligands decomposed H_2_O_2_ at a smaller rate. However, H_2_O_2_ decomposition by DFOB-altered fibers, from which 0.7 μmol g^–1^ Cr and 29 μmol g^–1^ Fe had been leached (Table 2, Table S5), was still larger than that by the MOPS-buffer. H_2_O_2_ decomposition by DTPA-altered and EDTA-altered fibers was comparable to the MOPS buffer (Figure 5a). DTPA and EDTA had removed larger amounts of Cr (1.9 and 2.2 μmol g^–1^, respectively) and Fe (36 and 40 μmol g^–1^, respectively) from the fibers during preconditioning compared to DFOB (Table 2 and Table S5).

In agreement with Walter et al.,^15,30^ the HO^•^ yield was considerably larger for blank-altered fibers (62%) than for DFOB-altered fibers (9%) (Figure 5b), from which Cr and Fe had been leached during fiber preconditioning (Table 2, Table S5). The HO^•^ yields of EDTA-altered fibers (14%) and DTPA-altered fibers (3%), from which even larger amounts of Cr and Fe had been leached during preconditioning than by DFOB (Table 2, Table S5), were in a similar low range as the HO^•^ yield of DFOB-altered fibers.

## Discussion

### Leaching of Cr and Its Potential to Contribute to the Carcinogenicity of Chrysotile Fibers

As hypothesized, addition of H_2_O_2_ strongly increased the leaching of Cr from pristine fibers at the physiological lung pH of 7.4 (to a similar extent as the strong Cr(III) chelators DTPA and EDTA, Figure 1) but also from weathered (preconditioned) chrysotile (Figure 2, Table 2). At circumneutral pH, H_2_O_2_ oxidizes the poorly soluble trivalent Cr to the well soluble hexavalent Cr oxyanion.^42,65,83,84^ Results from our LC-ICP-MS analyses demonstrated that the Cr solution speciation in leachates from chrysotile treatments with H_2_O_2_ at pH 7.4 (Figure 2) were governed by hexavalent Cr; according to our equilibrium predictions, Cr was predominantly present as the CrO_4_^2–^ and HCrO_4_^–^ species (Figure 3, Table S6). However, our LC-ICP-MS speciation analyses further demonstrated that in acidic chrysotile suspensions (pH 3.0) to which no H_2_O_2_ was added, Cr(III) readily leached from chrysotile (Figure 3, Figure S5). This suggests that the bulk-Cr in Shijiazhuang chrysotile is Cr(III), and that at pH 3.0 a fraction of this Cr was mobilized through proton-promoted dissolution. Considering these results, we propose that the observed dissolution of Cr from chrysotile in the presence of H_2_O_2_ at pH 7.4 (Figure 2) was caused by oxidation of bulk-Cr(III) on the fiber surface to Cr(VI), which subsequently entered into solution. The negative charge of the outermost Si layer of chrysotile at pH 7.4^13^ presumably facilitates the leaching of the negatively charged chromate oxyanions. Of note, Cr mobilization through complexation by the organic MOPS buffer at pH 7.4 is very improbable, as (1) MOPS did not mobilize Cr in the blank treatments (Figure 2), and (2) rapid mobilization of Cr from chrysotile by H_2_O_2_ was also observed in the absence of MOPS (Figure S1b, Table S4).

The expression of the anion transporter proteins SLC4A1 and SLC26A1 in cells of typical asbestos-burdened tissues and asbestos-related cancers (Figure 4a–c) suggests that leached Cr(VI) can readily be taken up into their intracellular compartment. Indeed, lung epithelial, lung cancer, mesothelial, and mesothelioma cells all showed rapid uptake of Cr(VI) across a wide concentration range (0.2 to 2000 μmol L^–1^) (Figure 4d). Mesothelial derived cells were at least as susceptible to Cr(VI) uptake as lung derived cells. As Cr(VI) compounds are established human carcinogens in the lung,^51,57^ the comparable Cr(VI) uptake kinetics in mesothelial derived cells could indicate that these cells might be similarly susceptible to Cr(VI)-mediated carcinogenesis. Apart from replicates at the highest tested Cr(VI) concentration (2000 μmol L^–1^), Cr strongly accumulated in all four investigated cell types up to a factor of almost 10 in relation to the Cr(VI) levels added to the cell media (Figure 4f), suggesting that Cr(VI) that had leached from chrysotile is likely to accumulate in cells of burdened tissues.

The cellular Cr(VI) uptake experiments (Figure 4d–f) support the notion that lung epithelial, lung cancer, mesothelial, and mesothelioma cells can take up Cr(VI) by a transporter-mediated process. The considerable decrease in intracellular Cr content (by up to a factor of 45, Figure 4e) in replicates in which the anion-transporter inhibitor DIDS was added to the cell media demonstrates a transporter-mediated Cr(VI) uptake in these cells. As DIDS decreased the intracellular Cr content by a higher than equimolar extent at the highest Cr(VI) spike-in concentration of 2000 μmol L^–1^ (Figure S8b), and as soluble Cr concentrations did not decrease with addition of DIDS to the cell media (Figure S10), Cr(VI) uptake inhibition by DIDS was consequently caused by transporter inhibition rather than by other possible effects such as precipitation or complex formation of DIDS and Cr(VI) and subsequent sequestering of Cr(VI) in the cell media. A transporter-mediated uptake of Cr(VI) in the examined cells is also suggested by the lower fraction of intracellular Cr contents in respect to the amount added in high Cr(VI) spike-in replicates (especially at 2000 μmol L^–1^) compared to lower Cr(VI) replicates (0.2, 2, and 20 μmol L^–1^), which presumably indicates saturation of Cr(VI) anion transporters at high Cr(VI) concentrations (Figure 4d,f). It is furthermore unlikely that the measured Cr(VI) uptake was caused by cytotoxicity with subsequent cell lysis, as incubation with the highest concentrations of Cr(VI) and both inhibitors did not result in increased cytotoxicity compared to untreated cells (Figure S9).

During frustrated phagocytosis of asbestos fibers and chronic inflammation, immune cells like alveolar macrophages and neutrophils increase the release of H_2_O_2_ into the extracellular environment of burdened tissues.^20,21^ The increased oxidative capacity in these tissues presumably promotes the oxidation of trivalent Cr on chrysotile fibers and consequently the leaching of Cr(VI). Additionally, it is conceivable that during asbestos-mediated cell death, phagolysosomal contents such as H_2_O_2_ are released and lead to a burst of Cr(VI) dissolution from internalized asbestos fibers into the extracellular compartment. As hexavalent chromium is a potent and genotoxic carcinogen,^45,51,57^ its rapid oxidative dissolution from chrysotile in the presence of H_2_O_2_ (Figures 2 and 3) and subsequent cellular uptake and accumulation (Figure 4) may contribute to the pathogenesis and/or progression of asbestos-associated malignant diseases. Furthermore, the increased expression of growth factors in chronically inflamed asbestos-burdened tissues^21^ can be expected to increase cellular proliferation rates and thereby enhance the promotion of initiated or pre-malignant cells that have acquired a mutation by fiber-leached Cr. Hence, chronically inflamed asbestos-burdened tissues constitute a cellular environment promotive of both the leaching of hexavalent Cr from chrysotile asbestos and of unlocking its carcinogenic potential, particularly its high genotoxicity in tumor initiation and progression processes.

In previous studies, trace metals (including Cr) on chrysotile surfaces were postulated as potential contributors to the carcinogenicity of the fibers.^38,62−64^ The results from our experiments combined with the efficient in vivo dissolution of Cr from intrapleurally administered chrysotile in rats^19,39^ support this hypothesis. The Cr content of chrysotile is typically in the lower g kg^–1^ range (in Shijiazhuang chrysotile 1.3–1.4 g kg^–1^, Table 1), which is strongly enriched relative to the bulk Cr content of the amphibole asbestos minerals amosite and crocidolite.^31^ Our findings support the notion that a fraction of this Cr may leach out of the fibers and be taken up by burdened tissues.

The applied H_2_O_2_ concentration at the beginning of the leaching experiments (3.3 g L^–1^ ≈ 100 mmol L^–1^) was approximately two orders of magnitude larger than the maximum extracellular H_2_O_2_ concentrations expected in pathophysiological conditions (up to 1 mmol L^–1^).^23^ The H_2_O_2_ concentration declined during the course of the leaching experiments due to degradation by chrysotile (Figure 5a, Walter et al.^30^). Nevertheless, the H_2_O_2_ concentration remained higher than concentrations to be expected in asbestos-burdened tissues.^23^ However, in a supplementary experiment, we demonstrated that H_2_O_2_ concentrations within the expected extracellular levels^23^ can mobilize Cr from chrysotile at pH 7.4 (Figure S4). Furthermore, considering that the residence time of chrysotile in burdened tissues will be orders of magnitude longer than the incubation times in the leaching experiments (2 weeks), mobilization may continue for a much larger time-span. Also, H_2_O_2_ concentrations in the direct vicinity of asbestos (e.g., during frustrated phagocytosis) might be considerably higher than averaged extracellular H_2_O_2_ concentrations under pathological conditions. Therefore, we postulate that Cr(VI) also leaches from chrysotile in vivo upon inhalation and subsequent instillation in burdened tissues.

Cr leaching was only marginally affected by preconditioning with blank and ligand solutions (Figure 2, Table 2). Hence, it can be concluded that not only pristine but also weathered chrysotile exhibits a high risk for H_2_O_2_-promoted Cr(VI) leaching once the fibers are inhaled. Surprisingly, Cr leached to almost the same extent from fibers pretreated with ligand solutions as from pristine and blank-altered fibers (Table 2). This was not observed for Fe and Ni, which were considerably depleted on the fiber surface after ligand treatments (Table S5). The cause for this divergent dissolution of Cr from chrysotile compared to other heavy metals is unclear but may relate to comparatively slow ligand-promoted dissolution kinetics of Cr^85^ or Cr equilibrium partitioning between fibers and the aqueous phase in the presence of ligands, favoring the aqueous phase to a lesser extent. The Cr present at the chrysotile surface after pretreatment could be rapidly mobilized through oxidative dissolution by H_2_O_2_. The cumulative amount of Cr mobilized after preconditioning and H_2_O_2_ treatment relates linearly with the cumulative amounts of mobilized Mg and Si (Figure S1c,d). Because in chrysotile Cr substitutes for Mg and the outer layer is a Mg layer,^1,31^ the linear regression with Mg goes through the origin, while the linear regression with Si has an intercept. These linear relations imply that, once the outer Mg layer had dissolved, Cr, Mg, and Si were liberated from the chrysotile structure in a fixed ratio, but during pretreatment, only a part of the liberated Cr at the surface had entered into solution.

### Contribution of Cr to Fiber-Mediated H_2_O_2_ Degradation and HO^•^ Generation

Preconditioning of chrysotile fibers with the chelators EDTA and DTPA did not deplete the fiber surfaces from reactive Cr (Table 2). However, the H_2_O_2_ decomposition rates in suspensions of EDTA-altered and DTPA-altered fibers were comparable to the rate in the MOPS-buffer control (Figure 5a). This suggests that the contribution of the remaining Cr on the fiber surface to H_2_O_2_ degradation is negligible, falsifying our hypothesis.

A possible explanation for the very low H_2_O_2_ decomposition by EDTA-altered and DTPA-altered fibers relative to DFOB-altered fibers is that EDTA and DTPA more effectively removed Fe from the fiber surfaces during preconditioning (Figure S3, Table S5). Walter et al.^30^ demonstrated that reactive Fe surface sites (especially Fe^3+^_tet_) are highly active in decomposing H_2_O_2_. So, a more effective depletion of these active sites may have rendered the fibers more inactive in decomposing H_2_O_2_.^30^ Furthermore, Walter et al. proposed a “remnant mode of H_2_O_2_ decomposition” for DFOB-altered chrysotile asbestos.^30^ Our experiments indicate that this remnant mode is related to remaining surface-exposed Fe^3+^_tet_ sites after pretreatment with DFOB rather than to other transition metals on fiber surfaces or magnetite impurities, supporting the hypothesis that Fe^3+^_tet_ is (on a molar basis) the most reactive species on chrysotile fibers in decomposing H_2_O_2_.^30^

The generation of HO^•^ radicals by fibers preconditioned with chelating ligands was strongly decreased in comparison to blank-altered fibers (Figure 5b). This resulted from the depletion of surface Fe^3+^_tet_ through complexation and mobilization by the ligands.^15,30^ The HO^•^ yields of fibers preconditioned with ligands varied between 3 and 14% for DTPA-altered and EDTA-altered fibers, respectively. Considering that the preconditioning of chrysotile fibers with DTPA did not deplete the fiber surfaces from reactive Cr (Table 2), the near-background HO^•^ yield of this fiber type demonstrates that a potential contribution of Cr to HO^•^ is negligible, which also falsifies our hypothesis. As Cr is the only Fenton-active metal in chrysotile apart from Fe (Table 1 and Valko et al.^56^), this conclusion supports the hypothesis that Fe^3+^_tet_ is the only relevant Fenton-active species on chrysotile fibers.^15,30^ The differences and variance in “remnant” HO^•^ yield for fibers preconditioned with ligands may result from differences in residual active Fe^3+^_tet_ surface sites related to differences in effectiveness of the applied ligands in complexing Fe^3+^_tet_ and from a heterogenous distribution of accessory Fenton-active magnetite phase impurities in the chrysotile material during the EPR spin trapping analyses.^24^

### Contribution of Ni to the Carcinogenicity of Chrysotile

Whereas in the absence of ligands and H_2_O_2_ dissolved Cr concentrations remained mostly below the limit of detection, Ni dissolved from pristine and preconditioned fiber surfaces to concentrations in the submicromolar range (below 0.5 μmol L^–1^) in blank solutions at pH 7.4 (Figure S6). Ni(II) does not participate in redox reactions with H_2_O_2_, and hence, the oxidative dissolution mechanisms observed for Cr(III) did not apply. Therefore, the presence of H_2_O_2_ did not consistently increase the leaching of Ni (Figure S6). Because oxidants like H_2_O_2_ do not enhance the leaching of Ni from chrysotile and the toxicity of Ni is less problematic as compared to Cr(VI) (weak mutagenicity, mostly dose-dependent carcinogenic effects like depletion of antioxidants and disruption of DNA repair mechanisms),^59−61,86^ the in vivo leaching of Ni from chrysotile presumably poses a lower carcinogenic risk than the leaching of Cr(VI). However, leached Ni may support the carcinogenic process in asbestos-burdened tissues via the aforementioned processes.

## Conclusions

Our results show that in the presence of H_2_O_2_, Cr rapidly leaches from chrysotile fibers by an oxidative dissolution mechanism in its hexavalent redox state. The fast leaching of Cr from intrapleurally administered chrysotile in rats^19,39^ has demonstrated that in vivo Cr can also rapidly dissolve from chrysotile. Considering our experimental results, oxidative dissolution of hexavalent chromium by elevated extracellular H_2_O_2_ concentrations in asbestos-burdened tissues may contribute to this efficient in vivo leaching of Cr.^20,21^ The observed expression of anion transporters and efficient uptake of Cr(VI) by mesothelial, lung epithelial, mesothelioma, and lung carcinoma cells and the strong accumulation of Cr in these cells indicate that Cr(VI) leached from chrysotile may easily reach the DNA of cells of typically asbestos-burdened tissues and hence support tumor initiation and progression processes. In a recent review and publications therein on the role of genotoxicity in asbestos-induced cancers,^87^ the potential contribution of Cr to the carcinogenicity of asbestos was not considered. However, based on our results combined with results from in vivo leaching studies,^19,39^ leaching of hexavalent Cr should indeed be considered a potential contributor to the genotoxicity and carcinogenicity of chrysotile asbestos. Chromium on chrysotile surfaces, however, does not appear to contribute to a relevant extent to fiber-mediated Haber–Weiss cycling. Consequently, Fe^3+^_tet_ can be discerned as the only relevant Fenton-reactive surface species on chrysotile at pH 7.4.^15,30^ The lower observed leaching of Ni compared to Cr and its dose-dependent carcinogenicity and weak mutagenicity^59−61^ indicate that Ni associated with chrysotile asbestos poses a lower risk than Cr does.

## Supporting Information Available

Figures presenting data on the potential leaching of Cr from magnetite mineral impurities in the Shijiazhuang chrysotile material, amplification efficiency of the applied qPCR method, Ni and Fe mobilization by ligands, Cr mobilization from chrysotile at pH 7.4 between 0.1 and 100 mmol L^–1^ H_2_O_2_, Cr mobilization from chrysotile at pH 3.0, Ni and Fe mobilization in the presence of H_2_O_2_ at pH 7.4, chromatograms of all LC-ICP-MS Cr speciation analyses, Cr(VI) uptake inhibition by NA at 2 μmol L^–1^ and by DIDS at 2000 μmol L^–1^ in mesothelial derived cells, cytotoxicity assays of the performed cellular Cr(VI) incubation experiments, measured Cr contents in cell media with different anion transporter inhibitors added, and H_2_O_2_ degradation in the experiment of Figure S1b; tables presenting raw data of main text and supplementary figures or tables, additional Cr speciation data and Cr leaching data in the absence of organic MOPS (or PIPPS) buffer (Table S4), metal and Si dissolution from chrysotile during fiber preconditioning (Table S5), equilibrium solution Cr speciation modeling of chrysotile suspensions at pH 7.4 (Table S6), and additional supplementary data to figures in the Supporting Information document (PDF)

## Abbreviations

BET: Brunauer, Emmet, Teller
DIDS: 4,4′-diisothiocyanostilbene-2,2′-disulfonic acid
DFOB: desferrioxamine-B
DMPO: 5–5′-dimethyl-1-pyrroline-*N*-oxide
DMPO/HO^•^: adduct of DMPO and HO^•^
DTPA: diethylenetriaminepentaacetic acid
EDTA: ethylenediaminetetraacetic acid
EGTA: ethylene glycol-bis(β-aminoethyl ether)-*N*,*N*,*N*′,*N*′-tetraacetic acid
EPR: electron paramagnetic resonance
Fe^3+^_tet_: ferric tetrahedral Fe
ICP-MS: inductively coupled plasma mass spectrometry
ICP-OES: inductively coupled plasma optical emission spectrometry
Ipp: intensity peak-to-peak
IS: ionic strength
LC-ICP-MS: liquid chromatography coupled to ICP-MS
MOPS: 3-(*N*-morpholino)propane sulfonic-acid
NA: niflumic acid
NAA: neutron activation analysis
p.a.: pro analysis quality of chemical reagents
PBS: phosphate buffered saline
PIPPS: 1,4-piperazinedipropanesulfonic acid
PP: polypropylene
PVDF: polyvinylidene fluoride
qPCR: quantitative polymerase chain reaction
RPM: rounds per minute
SDS: sodium dodecyl sulfate
SLC4A1: solute carrier family 4, anion exchanger, member 1
SLC26A1: solute carrier family 26, anion exchanger, member 1
Tris: tris(hydroxymethyl)aminomethane
UV–vis: ultra violet and visible light
WHO-IARC: World Health Organization, International Agency for Research on Cancer
WHO-IPCS: World Health Organization, International Program on Chemical Safety
XRD: X-ray diffraction

## References

[ref1] Catherine H.; Skinner W. Mineralogy of asbestos minerals. Indoor Built Environ. 2003, 12, 385–389. 10.1177/1420326X03037003.

[ref2] Oury T. D., Sporn T. A., Roggli V. L., and (Editors). (2014) Pathology of asbestos associated diseases, 10.1007/978-3-642-41193-9.

[ref3] Landrigan P. J. Asbestos - Still a carcinogen. N. Engl. J. Med. 1998, 338, 1618–1619. 10.1056/NEJM199805283382209. 9603801

[ref4] Frank A. The History of the Extraction and Uses of Asbestos. 1-7. 2005.

[ref5] Arsenic, metals, fibres and dusts. IARC Monogr. Eval. Carcinog. Risks Hum. 2012, 100, 219–294. PMC4781271 23189751

[ref6] Currie G. P.; Watt S. J.; Maskell N. A. An overview of how asbestos exposure affects the lung. BMJ 2009, 339, b3209 10.1136/bmj.b3209. 19703924

[ref7] WHO*,* International programme on chemical safety: Chrysotile asbestos*;* WHO 2014*, ISBN: 978 92 4 156481 6*.

[ref8] Nicholson W. J. The carcinogenicity of chrysotile asbestos - A review. Ind. Health. 2001, 39, 57–64. 10.2486/indhealth.39.57. 11341559

[ref9] Aust A. E.; Cook P. M.; Dodson R. F. Morphological and chemical mechanisms of elongated mineral particle toxicities. J. Toxicol. Env. Heal. B. 2011, 14, 40–75. 10.1080/10937404.2011.556046. PMC3118489 21534085

[ref10] Nishikawa K.; Takahashi K.; Karjalainen A.; Wen C. P.; Furuya S.; Hoshuyama T.; Todoroki M.; Kiyomoto Y.; Wilson D.; Higashi T.; Ohtaki M.; Pan G. W.; Wagner G. Recent Mortality from Pleural Mesothelioma, Historical Patterns of Asbestos Use, and Adoption of Bans: A Global Assessment. Environ. Health Perspect. 2008, 116, 1675–1680. 10.1289/ehp.11272. 19079719 PMC2599762

[ref11] Le G. V.; Takahashi K.; Park E. K.; Delgermaa V.; Oak C.; Qureshi A. M.; Aljunid S. M. Asbestos use and asbestos-related diseases in Asia: Past, present and future. Respirology 2011, 16, 767–775. 10.1111/j.1440-1843.2011.01975.x. 21449920

[ref12] Virta R. L. Worldwide asbestos supply and consumption trends from1900 through 2003*;* USGS Circular, 2006 1298.

[ref13] Bales R. C.; Morgan J. J. Surface-charge and adsorption properties of chrysotile asbestos in natural-waters. Environ. Sci. Technol. 1985, 19, 1213–1219. 10.1021/es00142a013. 22280140

[ref14] Evans B. W. The serpentinite multisystem revisited: Chrysotile is metastable. Int. Geol. Rev. 2004, 46, 479–506. 10.2747/0020-6814.46.6.479.

[ref15] Walter M.; Schenkeveld W.; Reissner M.; Gille L.; Kraemer S. M. The effect of pH and biogenic ligands on the weathering of chrysotile asbestos; the pivotal role of tetrahedral Fe in dissolution kinetics and radical formation. Chem. – Eur. J. 2019, 25, 3386–3300. 10.1002/chem.201804319. PMC6582442 30417458

[ref16] Gronow J. R. The dissolution of asbestos fibers in water. Clay Miner. 1987, 22, 21–35. 10.1180/claymin.1987.022.1.03.

[ref17] Thom J. G. M.; Dipple G. M.; Power I. M.; Harrison A. L. Chrysotile dissolution rates: Implications for carbon sequestration. Appl. Geochem. 2013, 35, 244–254. 10.1016/j.apgeochem.2013.04.016.

[ref18] Bales R. C.; Morgan J. J. Dissolution kinetics of chrysotile at pH 7 to 10. Geochim. Cosmochim. Acta 1985, 49, 2281–2288. 10.1016/0016-7037(85)90228-5.

[ref19] Morgan A.; Holmes A.; Gold C. Studies of the solubility of constituents of chrysotile asbestos in vivo using radioactive tracer techniques. Environ. Res. 1971, 4, 558–570. 10.1016/0013-9351(71)90016-8.

[ref20] Kamp D. W.; Graceffa P.; Pryor W. A.; Weitzman S. A. The role of free-radicals in asbestos-induced diseases. Free Radical Biol. Med. 1992, 12, 293–315. 10.1016/0891-5849(92)90117-Y. 1577332

[ref21] Kamp D. W.; Weitzman S. A. The molecular basis of asbestos induced lung injury. Thorax 1999, 54, 638–652. 10.1136/thx.54.7.638. 10377212 PMC1745526

[ref22] Hardy J. A.; Aust A. E. Iron in asbestos chemistry and carcinogenicity. Chem. Rev. 1995, 95, 97–118. 10.1021/cr00033a005.

[ref23] Sies H. Hydrogen peroxide as a central redox signaling molecule in physiological oxidative stress: Oxidative eustress. Redox. Biol. 2017, 11, 613–619. 10.1016/j.redox.2016.12.035. 28110218 PMC5256672

[ref24] Fubini B.; Mollo L.; Giamello E. Free radical generation at the solid/liquid interface in iron-containing minerals. Free Radical Res. 1995, 23, 593–614. 10.3109/10715769509065280. 8574353

[ref25] Fubini B.; Mollo L. Role of iron in the reactivity of mineral fibers. Toxicol. Lett. 1995, 82-83, 951–960. 10.1016/0378-4274(95)03531-1. 8597167

[ref26] Haber F.; Weiss J. Über die Katalyse des Hydroperoxydes. Naturwissenschaften 1932, 20, 948–950. 10.1007/BF01504715.

[ref27] De Laat J.; Gallard H. Catalytic decomposition of hydrogen peroxide by Fe(III) in homogeneous aqueous solution: Mechanism and kinetic modeling. Environ. Sci. Technol. 1999, 33, 2726–2732. 10.1021/es981171v.

[ref28] Gazzano E.; Turci F.; Foresti E.; Putzu M. G.; Aldieri E.; Silvagno F.; Lesci I. G.; Tomatis M.; Riganti C.; Romano C.; Fubini B.; Roveri N.; Ghigo D. Iron-loaded synthetic chrysotile: A new model solid for studying the role of iron in asbestos toxicity. Chem. Res. Toxicol. 2007, 20, 380–387. 10.1021/tx600354f. 17315889

[ref29] Xu A.; Huang X.; Lien Y.-C.; Bao L.; Yu Z.; Hei T. K. Genotoxic Mechanisms of Asbestos Fibers: Role of Extranuclear Targets. Chem. Res. Toxicol. 2007, 20, 724–733. 10.1021/tx600364d. 17447795

[ref30] Walter M.; Schenkeveld W. D. C.; Geroldinger G.; Gille L.; Reissner M.; Kraemer S. M. Identifying the reactive sites of hydrogen peroxide decomposition and hydroxyl radical formation on chrysotile asbestos surfaces. Part. Fibre Toxicol. 2020, 17, 3 10.1186/s12989-019-0333-1. 31959185 PMC6971994

[ref31] Bowes D. R.; Farrow C. M. Major and trace element compositions of the UICC standard asbestos samples. Am. J. Ind. Med. 1997, 32, 592–594. 10.1002/(SICI)1097-0274(199712)32:6<592::AID-AJIM3>3.0.CO;2-S. 9358914

[ref32] Schreier H. Asbestos in the natural environment; Elsevier: Amsterdam, 1989.

[ref33] Holmes A.; Morgan A.; Sandalls F. J. Determination of Iron, Chromium, Cobalt, Nickel, and Scandium in Asbestos by Neutron Activation Analysis. Am. Ind. Hyg. Assoc. J. 1971, 32, 281–286. 10.1080/0002889718506461. 5087573

[ref34] Bloise A.; Barca D.; Gualtieri A. F.; Pollastri S.; Belluso E. Trace elements in hazardous mineral fibres. Environ. Pollut. 2016, 216, 314–323. 10.1016/j.envpol.2016.06.007. 27289526

[ref35] Gualtieri A. F.; Lusvardi G.; Zoboli A.; Di Giuseppe D.; Lassinantti Gualtieri M. Biodurability and release of metals during the dissolution of chrysotile, crocidolite and fibrous erionite. Environ. Res. 2019, 171, 550–557. 10.1016/j.envres.2019.01.011. 30763876

[ref36] Morgan A.; Lally A. E.; Holmes A. Some observations on the distribution of trace metals in chrysotile asbestos. Ann. Occup. Hyg. 1973, 16, 231–240. 4804921 10.1093/annhyg/16.3.231

[ref37] Barbeau C.; Dupuis M.; Roy J. C. Metallic elements in crude and milled chrysotile asbestos from Quebec. Environ. Res. 1985, 38, 275–282. 10.1016/0013-9351(85)90091-X. 2998751

[ref38] Gross P.; deTreville R. T. P.; Tolker E. B.; Kaschak M.; Babyak M. A. Experimental Asbestosis. Arch. Environ. Health 1967, 15, 343–355. 10.1080/00039896.1967.10664930. 6035084

[ref39] Holmes A.; Morgan A. Leaching of Constituents of Chrysotile Asbestos in vivo. Nature 1967, 215, 441–442. 10.1038/215441b0. 6058318

[ref40] McClain C. N.; Fendorf S.; Webb S. M.; Maher K. Quantifying Cr(VI) Production and Export from Serpentine Soil of the California Coast Range. Environ. Sci. Technol. 2017, 51, 141–149. 10.1021/acs.est.6b03484. 27935688

[ref41] Oze C.; Fendorf S.; Bird D. K.; Coleman R. G. Chromium Geochemistry of Serpentine Soils. Int. Geol. Rev. 2004, 46, 97–126. 10.2747/0020-6814.46.2.97.

[ref42] Maurizio P.; Millero F. Chromium speciation in seawater: The probable role of hydrogen peroxide. Limnol. Oceanogr. 1990, 35, 730–736. 10.4319/lo.1990.35.3.0730.

[ref43] Rock M. L.; James B. R.; Helz G. R. Hydrogen Peroxide Effects on Chromium Oxidation State and Solubility in Four Diverse, Chromium-Enriched Soils. Environ. Sci. Technol. 2001, 35, 4054–4059. 10.1021/es010597y. 11686366

[ref44] Dayan A. D.; Paine A. J. Mechanisms of chromium toxicity, carcinogenicity and allergenicity: Review of the literature from 1985 to 2000. Hum. Exp. Toxicol. 2001, 20, 439–451. 10.1191/096032701682693062. 11776406

[ref45] Nickens K. P.; Patierno S. R.; Ceryak S. Chromium genotoxicity: a double-edged sword. Chem.-Biol. Interact. 2010, 188, 276–288. 10.1016/j.cbi.2010.04.018. 20430016 PMC2942955

[ref46] De Flora S.; Bagnasco M.; Serra D.; Zanacchi P. Genotoxicity of chromium compounds. A review. Mutat. Res. 1990, 238, 99–172. 10.1016/0165-1110(90)90007-X. 2407950

[ref47] Cieslak-Golonka M. Toxic and mutagenic effects of chromium(VI). A review. Polyhedron 1996, 15, 3667–3689. 10.1016/0277-5387(96)00141-6.

[ref48] Wei X.; Hu L.-L.; Chen M.-L.; Yang T.; Wang J.-H. Analysis of the Distribution Pattern of Chromium Species in Single Cells. Anal. Chem. 2016, 88, 12437–12444. 10.1021/acs.analchem.6b03810. 28193078

[ref49] Jennette K. W. Chromate metabolism in liver microsomes. Biol. Trace Elem. Res. 1979, 1, 55–62. 10.1007/BF02783843. 24276982

[ref50] Ottenwälder H.; Wiegand H. J.; Bolt H. M. Membrane permeability and intracellular disposition of 51Cr(VI) in human red blood cells. Toxicol. Environ. Chem. 1987, 14, 219–226. 10.1080/02772248709357205.

[ref51] Wang Y.; Su H.; Gu Y.; Song X.; Zhao J. Carcinogenicity of chromium and chemoprevention: a brief update. Onco Targets Ther 2017, Volume 10, 4065–4079. 10.2147/OTT.S139262. PMC5565385 28860815

[ref52] Cohen M. D.; Kargacin B.; Klein C. B.; Costa M. Mechanisms of Chromium Carcinogenicity and Toxicity. Crit. Rev. Toxicol. 1993, 23, 255–281. 10.3109/10408449309105012. 8260068

[ref53] Bal W.; Protas A. M.; Kasprzak K. S. Genotoxicity of metal ions: chemical insights. Met Ions Life Sci 2011, 8, 319–373. 21473386

[ref54] Zhitkovich A. Importance of Chromium–DNA Adducts in Mutagenicity and Toxicity of Chromium(VI). Chem. Res. Toxicol. 2005, 18, 3–11. 10.1021/tx049774+. 15651842

[ref55] Plaper A.; Jenko-Brinovec Š.; Premzl A.; Kos J.; Raspor P. Genotoxicity of Trivalent Chromium in Bacterial Cells Possible Effects on DNA Topology. Chem. Res. Toxicol. 2002, 15, 943–949. 10.1021/tx010096q. 12119005

[ref56] Valko M.; Morris H.; Cronin M. Metals, toxicity and oxidative stress. Curr. Med. Chem. 2005, 12, 1161–1208. 10.2174/0929867053764635. 15892631

[ref57] Arsenic, metals, fibres and dusts. IARC Monogr. Eval. Carcinog. Risks Hum. 2012, 100C, 147–168. PMC4781271 23189751

[ref58] Arsenic, metals, fibres and dusts. IARC Monogr. Eval. Carcinog. Risks Hum. 2012, 100C, 169–218. PMC4781271 23189751

[ref59] Kim H. S.; Kim Y. J.; Seo Y. R. An Overview of Carcinogenic Heavy Metal: Molecular Toxicity Mechanism and Prevention. J. Cancer Prev. 2015, 20, 232–240. 10.15430/JCP.2015.20.4.232. 26734585 PMC4699750

[ref60] Hartwig A.; Krüger I.; Beyersmann D. Mechanisms in nickel genotoxicity: the significance of interactions with DNA repair. Toxicol. Lett. 1994, 72, 353–358. 10.1016/0378-4274(94)90048-5. 8202952

[ref61] Cameron K. S.; Buchner V.; Tchounwou P. B. Exploring the Molecular Mechanisms of Nickel-Induced Genotoxicity and Carcinogenicity: A Literature Review. Environ. Health Rev. 2011, 26, 81–92. 10.1515/reveh.2011.012. PMC3172618 21905451

[ref62] Cralley L. J.; Keenan R. G.; Lynch J. R. Exposure to Metals in the Manufacture of Asbestos Textile Products. Am. Ind. Hyg. Assoc. J. 1967, 28, 452–461. 10.1080/00028896709342664. 6055841

[ref63] Cralley L. J.; Keenan R. G.; Kupel R. E.; Kinser R. E.; Lynch J. R. Characterization and Solubility of Metals Associated with Asbestos Fibers. Am. Ind. Hyg. Assoc. J. 1968, 29, 569–573. 10.1080/00028896809343057. 4302934

[ref64] Harington J. S.; Roe F. J. C. Studies of carcinogenesis of asbestos and their natural oils. Ann. N. Y. Acad. Sci. 1965, 132, 439–450. 10.1111/j.1749-6632.1965.tb41125.x. 5219566

[ref65] Baloga M. R.; Earley J. E. The Kinetics of the Oxidation of Cr(III) to Cr(VI) by Hydrogen Peroxide1. J. Am. Chem. Soc. 1961, 83, 4906–4909. 10.1021/ja01485a009.

[ref66] Walter M.; Geroldinger G.; Gille L.; Kraemer S. M.; Schenkeveld W. D. C. Soil-pH and cement influence the weathering kinetics of chrysotile asbestos in soils and its hydroxyl radical yield. J. Hazard. Mater. 2022, 431, 128068 10.1016/j.jhazmat.2021.128068. 35359096

[ref67] Daghino S.; Martino E.; Fenoglio I.; Tomatis M.; Perotto S.; Fubini B. Inorganic materials and living organisms: Surface modifications and fungal responses to various asbestos forms. Chem. – Eur. J. 2005, 11, 5611–5618. 10.1002/chem.200500046. 16021644

[ref68] Turci F.; Favero-Longo S. E.; Tomatis M.; Martra G.; Castelli D.; Piervittori R.; Fubini B. A biomimetic approach to the chemical inactivation of chrysotile fibres by lichen metabolites. Chem. – Eur. J. 2007, 13, 4081–4093. 10.1002/chem.200600991. 17295378

[ref69] Bhattacharya S.; John P. J.; Ledwani L. Bacterial Weathering of Asbestos. Silicon 2015, 7, 419–431. 10.1007/s12633-014-9260-9.

[ref70] Pecsok R. L.; Shields L. D.; Schaefer W. P. Complexes of Chromium(II) and (III) with Ethylenediaminetetraacetic Acid. Inorg. Chem. 1964, 3, 114–116. 10.1021/ic50011a024.

[ref71] Bucci R.; Magri A. L.; Napoli A. Chromium(III) Complexes with Diethylentriaminepentaacetic Acid. J. Coord. Chem. 1991, 24, 169–175. 10.1080/00958979109409459.

[ref72] Kandegedara A.; Rorabacher D. B. Noncomplexing tertiary amines as ″better″ buffers covering the range of pH 3-11 Temperature dependence of their acid dissociation constants. Anal. Chem. 1999, 71, 3140–3144. 10.1021/ac9902594. 21662904

[ref73] Chao C. C.; Aust A. E. Effect of long-term removal of iron from asbestos by desferrioxamine B on subsequent mobilization by other chelators and induction of DNA single-strand breaks. Arch. Biochem. Biophys. 1994, 308, 64–69. 10.1006/abbi.1994.1009. 8311475

[ref74] Weitzman S. A.; Chester J. F.; Graceffa P. Binding of deferoxamine to asbestos fibers in vitro and in vivo. Carcinogenesis 1988, 9, 1643–1645. 10.1093/carcin/9.9.1643. 3409466

[ref75] Duckworth O. W.; Akafia M. M.; Andrews M. Y.; Bargar J. R. Siderophore-promoted dissolution of chromium from hydroxide minerals. Environ. Sci.: Processes Impacts 2014, 16, 1348–1359. 10.1039/C3EM00717K. 24683601

[ref76] Zhao G.; Chasteen N. D. Oxidation of Good’s buffers by hydrogen peroxide. Anal. Biochem. 2006, 349, 262–267. 10.1016/j.ab.2005.10.005. 16289439

[ref77] Enrico Favero-Longo S.; Turci F.; Tomatis M.; Compagnoni R.; Piervittori R.; Fubini B. The Effect of Weathering on Ecopersistence, Reactivity, and Potential Toxicity of Naturally Occurring Asbestos and Asbestiform Minerals. J. Toxicol. Environ. Health, Part A 2009, 72, 305–314. 10.1080/15287390802529864. 19184746

[ref78] Schelch K.; Hoda M. A.; Klikovits T.; Münzker J.; Ghanim B.; Wagner C.; Garay T.; Laszlo V.; Setinek U.; Dome B.; Filipits M.; Pirker C.; Heffeter P.; Selzer E.; Tovari J.; Torok S.; Kenessey I.; Holzmann K.; Grasl-Kraupp B.; Marian B.; Klepetko W.; Berger W.; Hegedus B.; Grusch M. Fibroblast growth factor receptor inhibition is active against mesothelioma and synergizes with radio- and chemotherapy. Am. J. Respir. Crit. Care Med. 2014, 190, 763–772. 10.1164/rccm.201404-0658OC. 25188816

[ref79] Pirker C.; Bilecz A.; Grusch M.; Mohr T.; Heidenreich B.; Laszlo V.; Stockhammer P.; Lötsch-Gojo D.; Gojo J.; Gabler L.; Spiegl-Kreinecker S.; Dome B.; Steindl A.; Klikovits T.; Hoda M. A.; Jakopovic M.; Samarzija M.; Mohorcic K.; Kern I.; Kiesel B.; Brcic L.; Oberndorfer F.; Müllauer L.; Klepetko W.; Schmidt W. M.; Kumar R.; Hegedus B.; Berger W. Telomerase Reverse Transcriptase Promoter Mutations Identify a Genomically Defined and Highly Aggressive Human Pleural Mesothelioma Subgroup. Clin. Cancer Res. 2020, 26, 3819–3830. 10.1158/1078-0432.CCR-19-3573. 32317288

[ref80] Wehner F.; Rosin-Steiner S.; Beetz G.; Sauer H. The anion transport inhibitor DIDS increases rat hepatocyte K+ conductance via uptake through the bilirubin pathway. J. Physiol. 1993, 471, 617–635. 10.1113/jphysiol.1993.sp019919. 8120826 PMC1143980

[ref81] Ben-Hail D.; Shoshan-Barmatz V. VDAC1-interacting anion transport inhibitors inhibit VDAC1 oligomerization and apoptosis. Biochim. Biophys. Acta - Mol. Cell Res. 2016, 1863, 1612–1623. 10.1016/j.bbamcr.2016.04.002. 27064145

[ref82] Parkhurst D. L., Appelo C. A. J. (1999.) User’s Guide to PHREEQC (version 2). A Computer Program for Speciation, Batch Reaction, One-Dimensional Transport, and Inverse Geochemical Calculations; U.S. Geological Survey Water-Resources Investigations Report pp. 99–4259.

[ref83] Luo Z.; Chatterjee N. Kinetics of oxidation of Cr(III)-organic complexes by H2O2. Chem. Speciation Bioavailability 2010, 22, 25–34. 10.3184/095422909X12548400846521.

[ref84] Maurizio P.; Gennari F.; Campanella L.; Millero F. The effect of organic compounds in the oxidation kinetics of Cr(III) by H2O2. Geochim. Cosmochim. Acta 2008, 72, 5692–5707.

[ref85] Saad E. M.; Sun J.; Chen S.; Borkiewicz O. J.; Zhu M.; Duckworth O. W.; Tang Y. Siderophore and Organic Acid Promoted Dissolution and Transformation of Cr(III)-Fe(III)-(oxy)hydroxides. Environ. Sci. Technol. 2017, 51, 3223–3232. 10.1021/acs.est.6b05408. 28218537

[ref86] Permenter M. G.; Lewis J. A.; Jackson D. A. Exposure to nickel, chromium, or cadmium causes distinct changes in the gene expression patterns of a rat liver derived cell line. PLoS One 2011, 6, e27730 10.1371/journal.pone.0027730. 22110744 PMC3218028

[ref87] Barlow C. A.; Lievense L.; Gross S.; Ronk C. J.; Paustenbach D. J. The role of genotoxicity in asbestos-induced mesothelioma: an explanation for the differences in carcinogenic potential among fiber types. Inhalation Toxicol. 2013, 25, 553–567. 10.3109/08958378.2013.807321. 23905972

