# Supplementary material for: The Potential Contribution of Hexavalent Chromium to the Carcinogenicity of Chrysotile Asbestos
Source: Chem Res Toxicol. 2022 Nov 21;35(12):2335–47. doi: 10.1021/acs.chemrestox.2c00314 (PMC9768810; doi:10.1021/acs.chemrestox.2c00314)
Supplement: Supplementary file 1 — tx2c00314_si_001.pdf [file tx2c00314_si_001.pdf]

Supplementary information: The potential contribution of hexavalent chromium to the carcinogenicity of chrysotile asbestos

Authors: Martin Walter [†], Walter D.C. Schenkeveld [†*‖], Maura Tomatis [⊥ꜰ], Karin Schelch [‡], Barbara Peter-Vörösmarty [‡], Gerald Geroldinger [§], Lars Gille [§], Maria C. Bruzzoniti [#], Francesco Turci [#ꜰ], Stephan M. Kraemer [†], Michael Grusch [‡]

[†] Department of Environmental Geosciences, University of Vienna, Althanstraße 14 (UZA II), 1090 Vienna, Austria

[⊥] Department of Veterinary Sciences, University of Torino, L.go Paolo Braccini, 2, 10095, Grugliasco (TO), Italy

[ꜰ] "G.Scansetti" Interdepartmental Center for Studies of Asbestos and Other Toxic Particulates, Via Pietro Giuria, 7, 10125, Torino, Italy

[‡] Center for Cancer Research, Medical University of Vienna, Borschkegasse 8a, 1090 Vienna, Austria

[§] Institute of Pharmacology and Toxicology, University of Veterinary Medicine, Vienna Veterinärplatz 1, 1210 Vienna, Austria

[#] Department of Chemistry, University of Torino, Via Pietro Giuria, 7, 10125, Torino, Italy

[*] Email: walter.schenkeveld@wur.nl,
[‖] Present address: Soil Chemistry and Chemical Soil Quality Group, Department of Environmental Sciences, Wageningen University, Droevendaalsesteeg 3A (Lumen building), 6708 PB Wageningen, the Netherlands

Table of contents:

	Page
**Discussion on Cr leaching from mineral impurities**	**S3**
**Supplementary-Figure 1:** Cr leaching from mineral impurities	**S7**
**Supplementary-Figure 2:** Amplification efficacy of the applied qPCR method	**S9**
**Supplementary-Figure 3:** Ni and Fe mobilization from chrysotile by ligands at pH 7.4	**S10**
**Supplementary-Figure 4:** Cr leaching from chrysotile at pH 7.4 at different H_2O_2 levels	**S11**
**Supplementary-Figure 5:** Cr leaching from chrysotile at pH 3.0	**S12**
**Supplementary-Figure 6:** Ni leaching from chrysotile in the presence of H_2O_2 at pH 7.4	**S13**
**Supplementary-Figure 7:** LC-ICP-MS chromatograms of the Cr speciation analyses	**S14**

Supplementary-Figure 8: Effects of anion transporter inhibitors on cellular Cr uptake S19
Supplementary-Figure 9: Cytotoxicity assessments in the Cr(VI) uptake experiment S20
Supplementary-Figure 10: Potential interference of DIDS & NA with Cr(VI) in cellular media S21
Supplementary-Figure 11: H_2O_2 degradation in the experiment of Supplementary-Figure 1b S22
Supplementary-Table 1: List of the cell types used in the study S23
Supplementary-Table 2: Data to main text Figure 1 S25
Supplementary-Table 3: Data to main text Figure 2 S26
Supplementary-Table 4: Data to main text Figure 3 & additional Cr speciation data S27
Supplementary-Table 5: Mg, Si, Fe and Ni removal during and after preconditioning S28
Supplementary-Table 6: Cr speciation equilibrium modelling at pH 7.4 S29
Supplementary-Table 7: Data to main text Figure 4 S30
Supplementary-Table 8: Data to main text Figure 5 S33
Supplementary-Table 9: Efficacy of the HNO_3 fiber digestion of chrysotile fibers S34
Supplementary-Table 10: Equilibrium modelling of Cr leached in Supplementary-Figure 1b S35
Supplementary-Table 11: Data to Supplementary-Figure 1 S36
Supplementary-Table 12: Data to Supplementary-Figure 2 S38
Supplementary-Table 13: Data to Supplementary-Figure 3 S39
Supplementary-Table 14: Data to Supplementary-Figure 4 S40
Supplementary-Table 15: Data to Supplementary-Figure 5 S41
Supplementary-Table 16: Data to Supplementary-Figure 6 S42
Supplementary-Table 17: Data to Supplementary-Figure 8 S44
Supplementary-Table 18: Data to Supplementary-Figure 10 S45
Supplementary-Table 19: Data to Supplementary-Figure 11 S46

Cr leaching from mineral impurities:

Cr is an elemental component of chrysotile [1]. However, the Shijiazhuang chrysotile material also contains brucite (4.5±2.1 %), talc (3.4±2.0 %), chlorite (2.4±2.9 %) and magnetite (1.5±0.2 %) [2], all of which can contain Cr and may therefore contribute to Cr dissolution from Shijiazhuang chrysotile. However, it is unlikely that brucite impurities have a larger Cr content than the brucite layers of chrysotile. Furthermore, talc and chlorite in serpentinites typically have a similar or lower Cr content than Shijiazhuang chrysotile [3-6]. Therefore, these mineral impurities are not thought to significantly contribute to overall Cr dissolution.

The Cr content of magnetite in serpentinites may vary considerably, from levels comparable to serpentine minerals [6] to considerably higher levels as e.g. observed in the Cogne (Italy) serpentinite: up to 17 wt.% Cr_2O_3 [7]. Hence, magnetite could potentially contribute substantially to Cr leaching from serpentinites. Therefore, the origin of Cr mobilized from the Shijiazhuang chrysotile material was further examined.

The examination included three analyses to investigate whether the leached Cr predominantly originated from chrysotile or from magnetite:

1) A 1.4 mol L^{-1} HNO_3-extraction was done at a fiber-to-solution ratio of 5 g L^{-1}, for 2 weeks at 50°C in 50 mL centrifugation tubes (Bio Greiner) in a horizontal shaker at 120 RPM. The extraction was designed to dissolve the total Mg content of the Shijiazhuang chrysotile fibers as well as the trace metals these Mg layers bear. The dissolved Mg content proved almost identical to the total Mg content determined by fusion digestion of Shijiazhuang chrysotile (Table 1, Supplementary-Table 9), indicating that the HNO_3 extraction effectively dissolved the Mg layers of the fibers. Also the dissolved Cr content in the HNO_3 extract corresponded well with the total Cr content determined by fusion digestion and NAA of Shijiazhuang chrysotile (Supplementary-Figure 1a). However, the dissolved Fe content in the HNO_3 extract was lower than the total Fe content of Shijiazhuang chrysotile determined by NAA; approximately 67% (Supplementary-Figure 1a). This fraction closely corresponds with the Fe fraction

incorporated in the chrysotile, as determined by Mössbauer spectroscopy (Table 1, Supplementary-Figure 1a). This suggests that the magnetite grains in the Shijiazhuang chrysotile material did not significantly dissolve during the HNO_3 extraction (fine magnetite grains were in fact still visible after the extraction). Indeed, natural magnetite is known to be highly stable towards acids; the residence time of such magnetite specimens at pH 1 is for example estimated to be in the tens of thousands years [8]. Furthermore, the HNO_3 extract suggests that most of the Cr was incorporated in chrysotile, as ≈ 100% of the Cr bulk was extracted with HNO_3. Therefore, it is unlikely that magnetite in Shijiazhuang chrysotile has an elevated Cr content and acts as an important source for Cr mobilization.

2.) Cr leaching from 1 g L^{-1} pristine and blank-altered fibers by H_2O_2 (3.3 g L^{-1} starting concentration) was examined in a 0.1 mol L^{-1} NaOH solution (in which Shijiazhuang chrysotile is insoluble [2]) for the same reaction times as applied in pH 7.4 leaching studies. Cr leaching was observed from blank-altered fibers, but not from pristine fibers (Supplementary-Figure 1b). The outermost Mg layer had dissolved during the preconditioning of blank-altered fibers (Supplementary-Table 5). This presumably led to precipitation or adsorption of its Cr(III) content onto the fiber surface (Figure 3, Supplementary-Table 6). As there is no indication of dissolution of magnetite impurities during the fiber preconditioning at pH 7.4 [8], the Cr mobilized from blank-altered fibers (Supplementary-Figure 1b) presumably corresponds with the precipitated/adsorbed Cr originating from the outermost Mg layers of the chrysotile fibers, and not from magnetite. H_2O_2 degradation data to this experiment can be found in Supplementary-Figure 11.

3.) Cumulatively dissolved Cr concentrations during preconditioning and the subsequent leaching experiments were correlated with cumulatively dissolved main elemental components of chrysotile (Mg and Si). The correlations proved to be linear for both Mg and Si (Supplementary Figure 1c and 1d, respectively). The linear relation between cumulatively dissolved Cr and Mg concentrations had no intercept, whereas the linear relation between cumulatively dissolved Cr and Si concentrations did: at 1.97 µmol g^{-1} Cr. These relations further support that Cr is predominantly released from the Mg layers of chrysotile fibers.

In conclusion: although a contribution from magnetite phase impurities to Cr leaching cannot be excluded, it is unlikely to be substantial.

References:

(1) Bowes, D. R., and Farrow, C. M. (1997) Major and trace element compositions of the UICC standard asbestos samples. *Am. J. Ind. Med. 32*, 592-594.

(2) Walter, M., Schenkeveld, W., Reissner, M., Gille, L., and Kraemer, S. M. (2019) The effect of pH and biogenic ligands on the weathering of chrysotile asbestos; the pivotal role of tetrahedral Fe in dissolution kinetics and radical formation. *Chem. Eur. J. 25*, 3386 – 3300.

(3) Debret, B., Andreani, M., Godard, M., Nicollet, C., Schwartz, S., and Lafay, R. (2013) Trace element behavior during serpentinization/de-serpentinization of an eclogitized oceanic lithosphere: A LA-ICPMS study of the Lanzo ultramafic massif (Western Alps). *Chem. Geol. 357*, 117-133.

(4) Tzamos, E., Bussolesi, M., Grieco, G., Marescotti, P., Crispini, L., Kasinos, A., Storni, N., Simeonidis, K., and Zouboulis, A. (2020) Mineralogy and Geochemistry of Ultramafic Rocks from Rachoni Magnesite Mine, Gerakini (Chalkidiki, Northern Greece). *Minerals 10*, 934.

(5) Yalçin, H., and Bozkaya, Ö. (2006) Mineralogy and Geochemistry of Paleocene Ultramafic- and Sedimentary-Hosted Talc Deposits in the Southern Part of the Sivas Basin, Turkey. *Clays Clay Miner. 54*, 333-350.

(6) Gahlan, H. A., Arai, S., Ahmed, A. H., Ishida, Y., Abdel-Aziz, Y. M., and Rahimi, A. (2006) Origin of magnetite veins in serpentinite from the Late Proterozoic Bou-Azzer ophiolite, Anti-Atlas, Morocco: An implication for mobility of iron during serpentinization. *Journal of African Earth Sciences 46*, 318-330.

(7) Carbonin, S., Martin, S., Tumiati, S., and Rossetti, P. (2015) Magnetite from the Cogne serpentinites (Piemonte ophiolite nappe, Italy). Insights into seafloor fluid–rock interaction. *Eur. J. Mineral. 27*, 31-50.

(8) White, A. F., Peterson, M. L., and Hochella, M. F. (1994) Electrochemistry and dissolution kinetics of magnetite and ilmenite. *Geochim. Cosmochim. Acta 58*, 1859-1875.

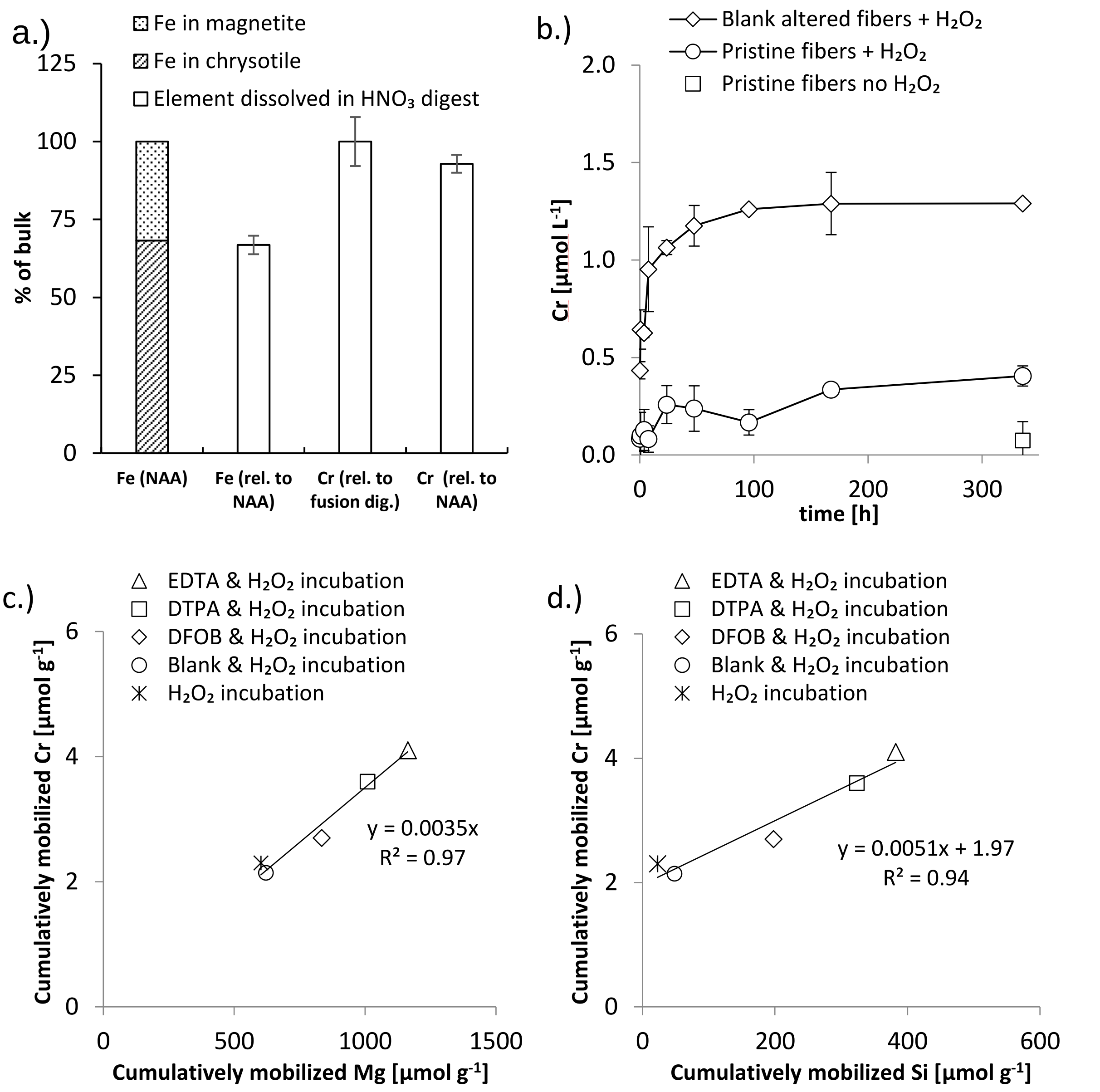

Supplementary-Figure 1: Panel a – Extraction efficacy for Fe and Cr in a HNO_3 fiber extraction (5 g L^{-1} pristine chrysotile extraction in 1.4 mol L^{-1} HNO_3 at 50°C for 2 weeks; this extraction dissolves ≈ 100% of the Mg content of the chrysotile fibers (Supplementary-Table 9)) relative to the total bulk-Cr and -Fe in Shijiazhuang chrysotile as determined by fusion digestions and/or a neutron activation analysis (NAA) (Table 1). The Fe fractions in magnetite and chrysotile were determined by Mössbauer spectroscopy (Table 1). The NAA was used to determine the total bulk-Fe as this method does not rely on a digestion of the Shijiazhuang chrysotile material. Panel b - Cr leaching from pristine and blank-altered chrysotile fibers (1 g L^{-1}) by 3.3 g L^{-1} H_2O_2 (starting concentration) under alkaline conditions (0.1

mol L^{-1} NaOH) at which chrysotile does not dissolve. Geochemical equilibrium modelling suggests that the leached Cr from blank-altered fibers was – similarly as for the Cr leaching experiments of Figure 2 – Cr(VI) (Supplementary-Table 10). Panel c & d - Cumulatively mobilized Cr concentrations in different fiber treatments at pH 7.4 as a function of the cumulatively mobilized Mg (Panel c) and Si (Panel d) concentration. Error bars indicate standard deviations (Panel a: n = 3, Panel b-d: n = 2). Data presented in this Figure are reported in Supplementary-Table 11.

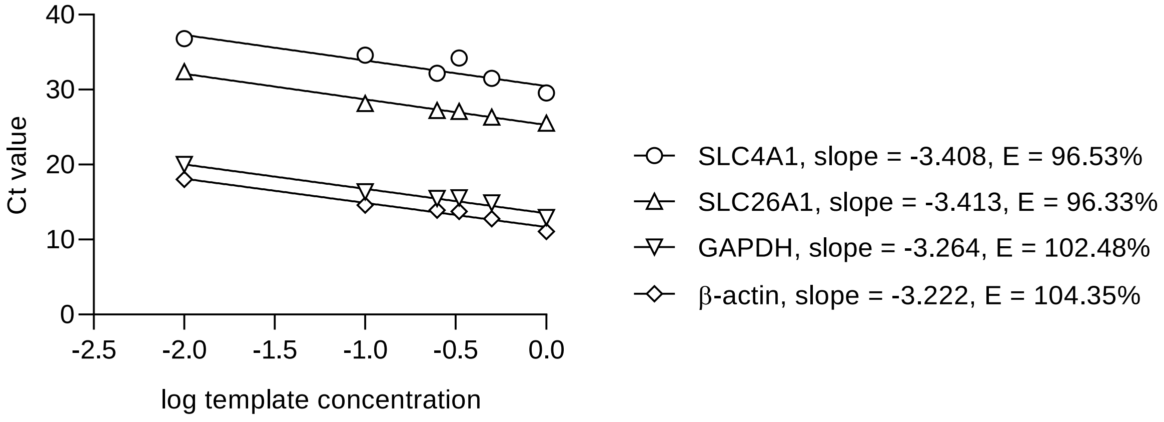

Supplementary-Figure 2: PCR amplification efficiencies of the primer pairs used. Dilution series of samples were subjected to qPCR and Ct values were plotted against the log10 of template concentration (undiluted sample set as 1). Linear regression was calculated with Graphpad Prism and % PCR efficiency was calculated as $E = -1+10^{(-1/slope)} \times 100$. Data presented in this Figure are reported in Supplementary-Table 12.

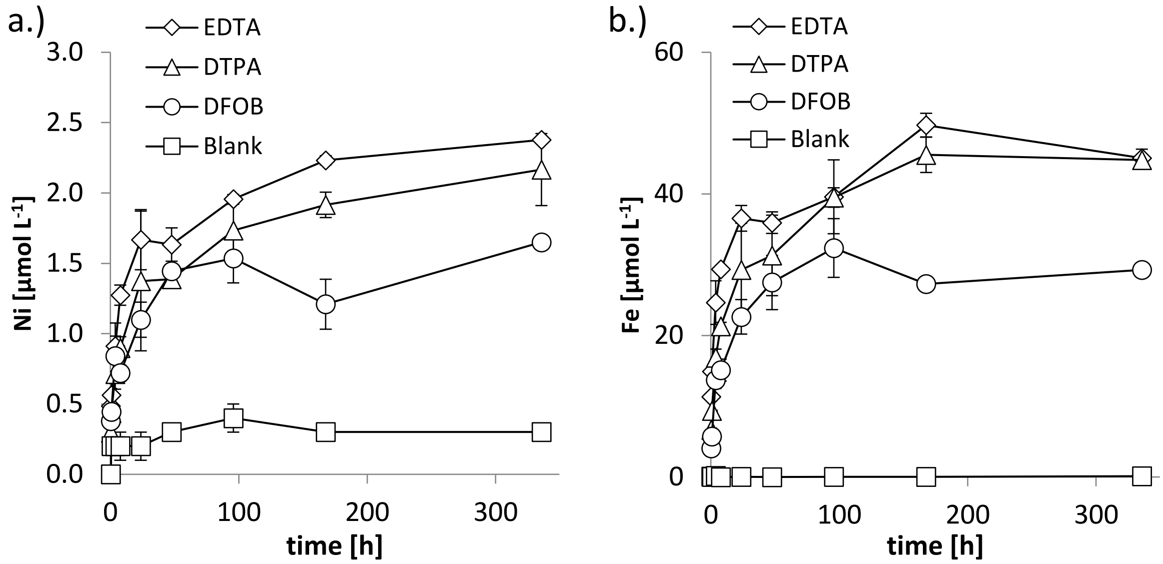

Supplementary-Figure 3: Ni (Panel a) and Fe (Panel b) concentrations mobilized from 1 g L^{-1} pristine fibers at pH 7.4 by 1 mmol L^{-1} of the chelators DTPA, EDTA and DFOB and in the absence of ligands (Blank). Error bars indicate standard deviations (n = 2). Data to this Figure are presented in Supplementary Table 13. The data for DFOB and blank Fe mobilization were taken from Walter et al. (2019) (Reference No. 15 in the main text).

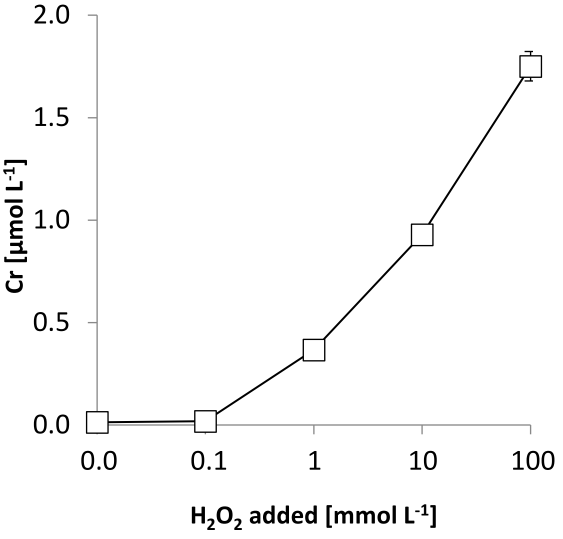

Supplementary-Figure 4: Mobilized Cr concentrations in 1 g L^{-1} chrysotile fiber suspensions buffered at pH 7.4 after 168 hours of incubation as a function the applied H_2O_2 concentrations (0, 0.1, 1, 10 and 100 $mmoL^{-1}$ - starting concentrations). Contrary to all other experiments, this experiment was conducted at 10 times lower NaCl background concentration (suprapur quality, Sigma) to facilitate ultra-trace metal analysis. At 0 mmol L^{-1} H_2O_2, 13.2 nmol L^{-1} Cr with a standard deviation of 2.9 nmol L^{-1} were measured, whereas at 0.1 mmol L^{-1} H_2O_2, 19.2 nmol L^{-1} Cr with a standard deviation of 0.5 nmol L^{-1} were measured. Error bars indicate standard deviations (n = 2). Data to this Figure are presented in Supplementary Table 14.

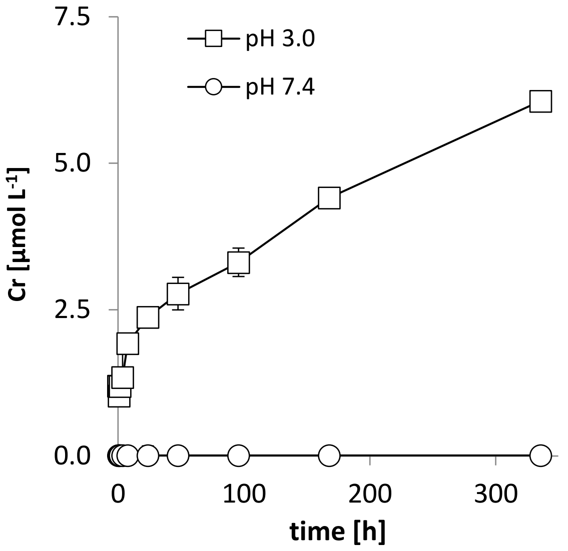

Supplementary-Figure 5: Cr mobilization in 1 g L^{-1} chrysotile suspensions at pH 3.0 ± 0.3 (PIPPS) and 7.4 ± 0.3 (MOPS) in absence of chromium chelators and H_2O_2. Error bars indicate standard deviations (n = 2). Data to this Figure are presented in Supplementary Table 15.

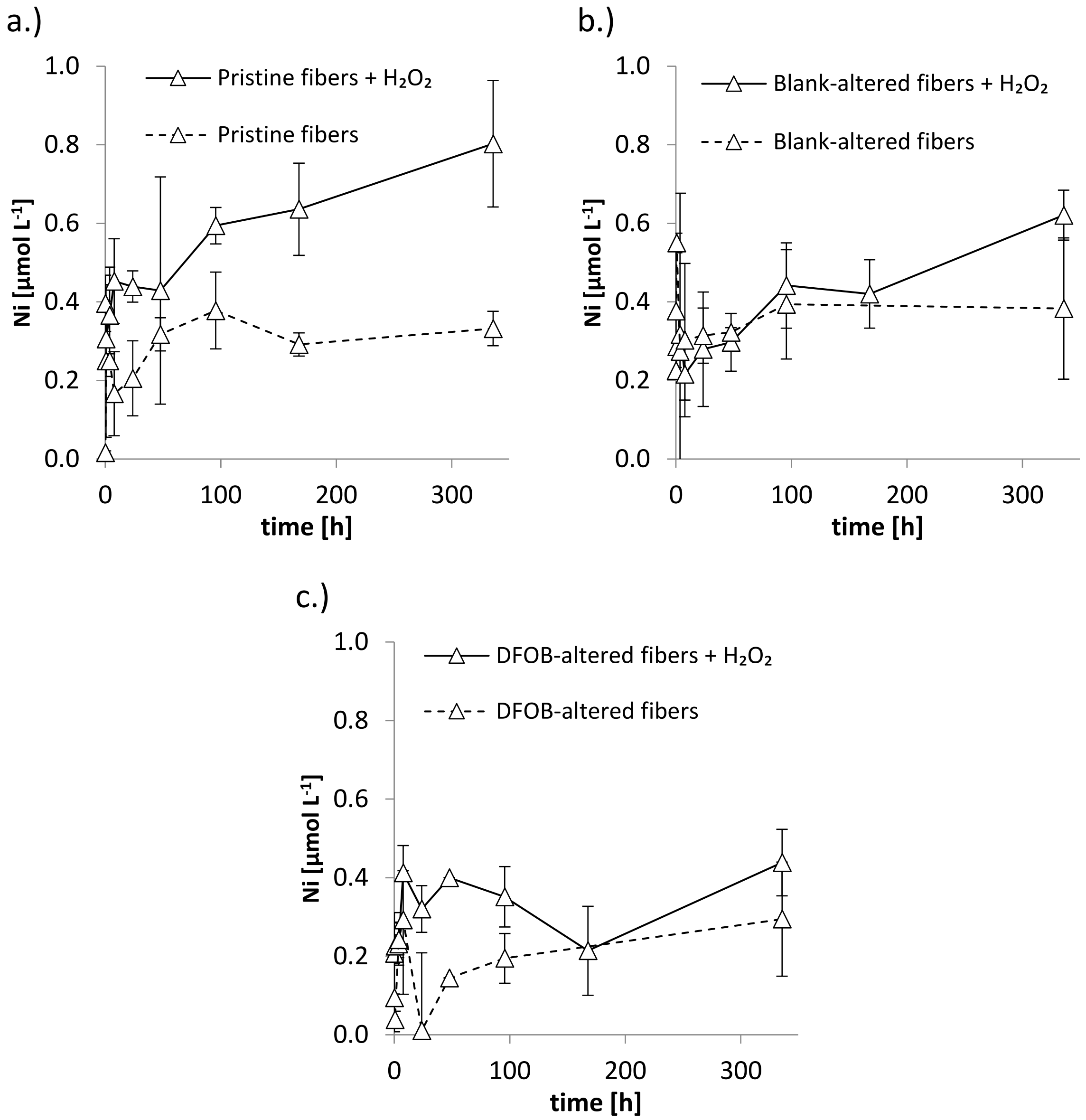

Supplementary-Figure 6: Ni concentrations mobilized from 1 g L^{-1} pristine (Panel a), blank-altered (Panel b) and DFOB-altered fibers (Panel c) as a function of time at pH 7.4, in the presence or absence of 3.3 g L^{-1} H_2O_2 (starting concentration). Error bars indicate standard deviations (n = 2). Data to this Figure are presented in Supplementary-Table 16.

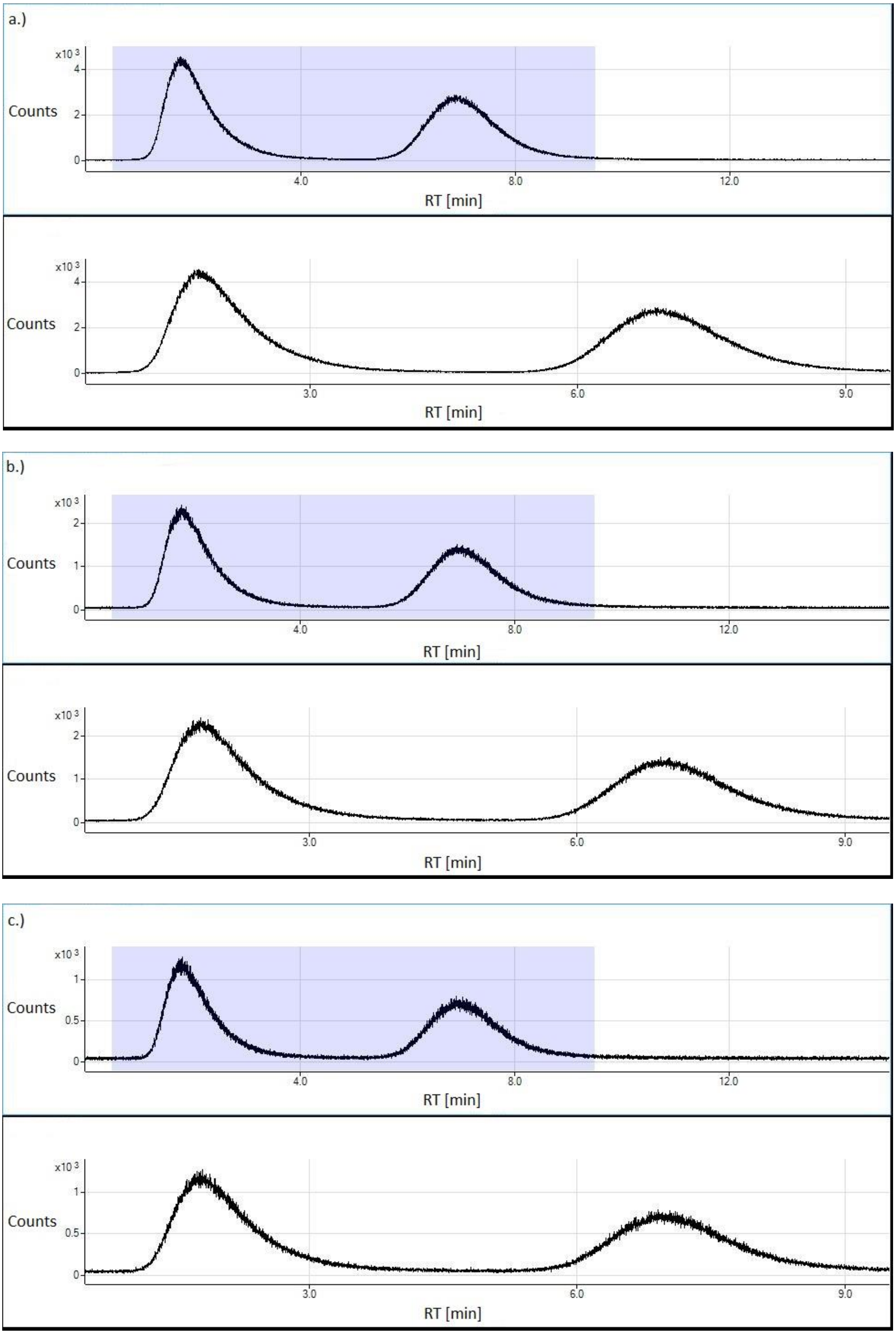
a.)
x10 3
Counts
RT [min]
b.)
c.)

d.)

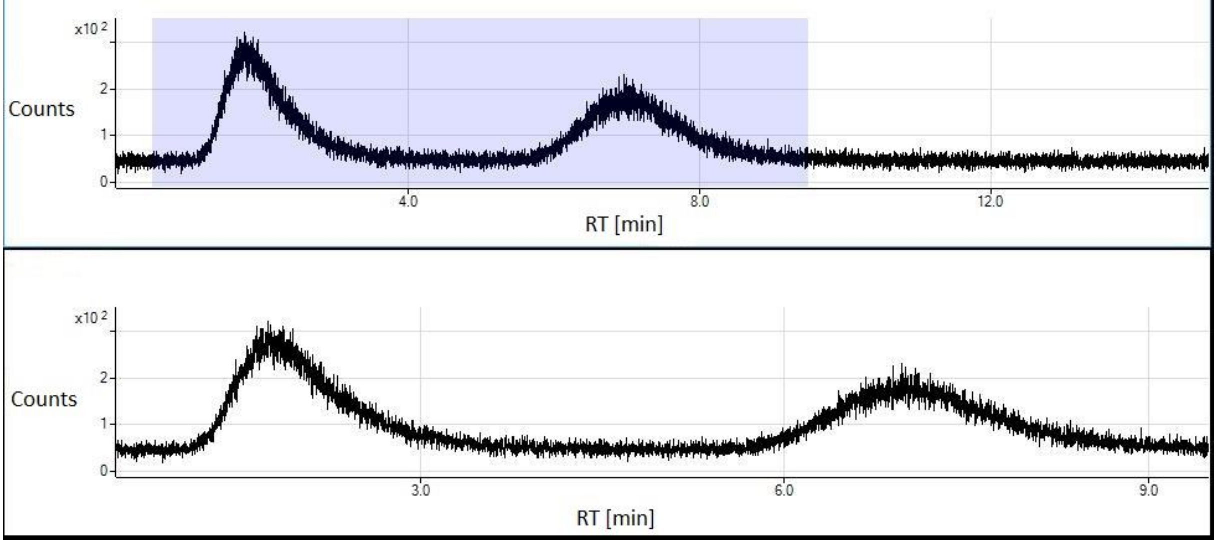

x10 2
2
1
0
Counts
4.0
8.0
12.0
RT [min]
x10 2
2
1
0
Counts
3.0
6.0
9.0
RT [min]

e1.)

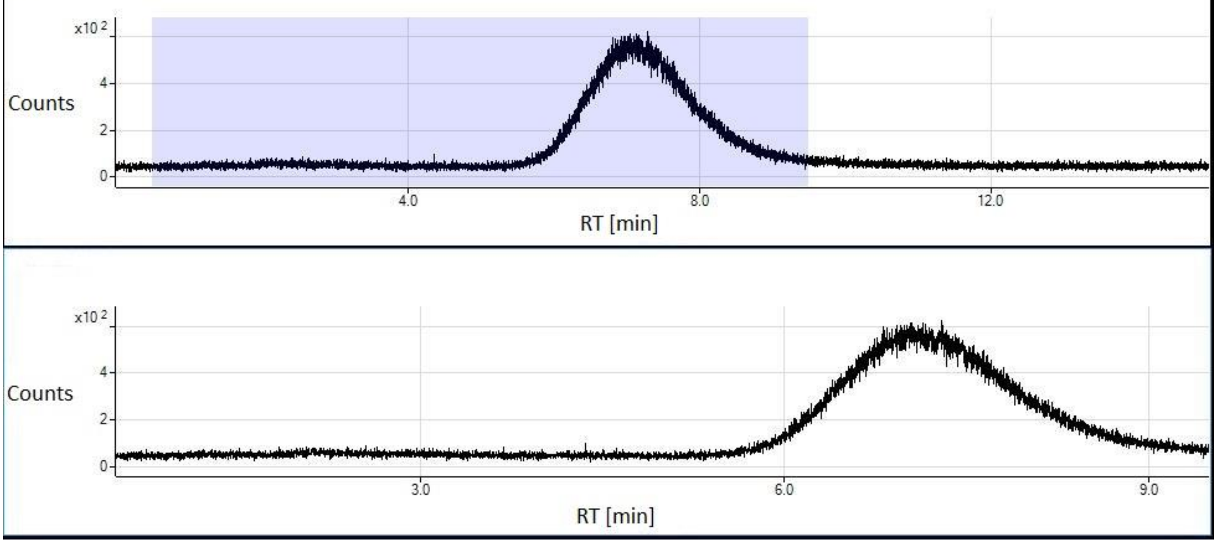

x10 2
4
2
0
Counts
4.0
8.0
12.0
RT [min]
x10 2
4
2
0
Counts
3.0
6.0
9.0
RT [min]

e2.)

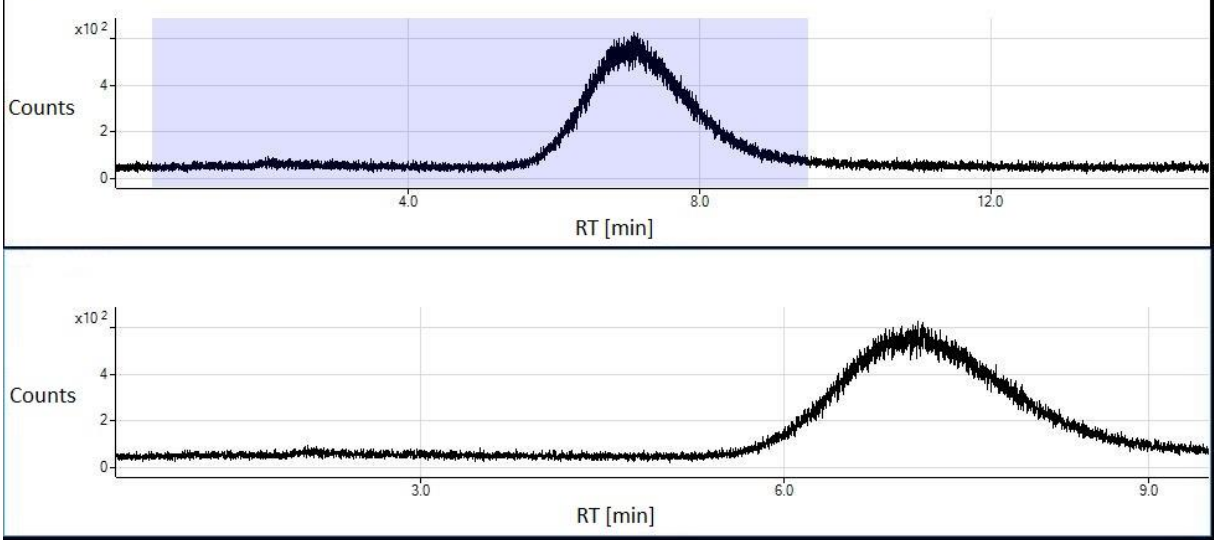

x10 2
4
2
0
Counts
4.0
8.0
12.0
RT [min]
x10 2
4
2
0
Counts
3.0
6.0
9.0
RT [min]

f1.)

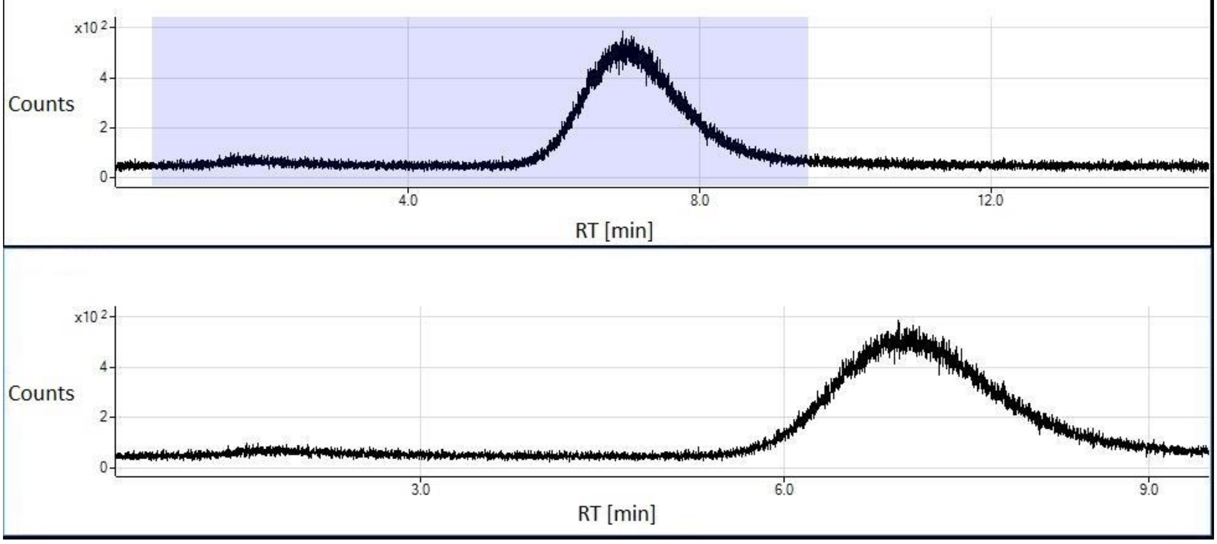
x10 2
Counts
0
2
4
4.0
8.0
12.0
RT [min]
x10 2
Counts
0
2
4
3.0
6.0
9.0
RT [min]

f2.)

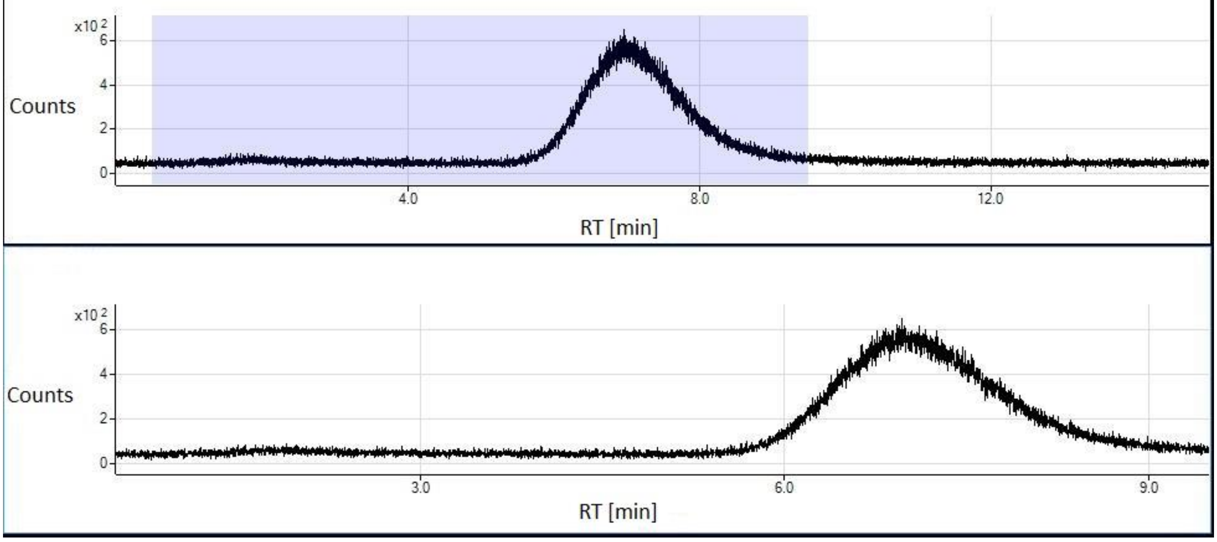
x10 2
Counts
0
2
4
6
4.0
8.0
12.0
RT [min]
x10 2
Counts
0
2
4
6
3.0
6.0
9.0
RT [min]

g1.)

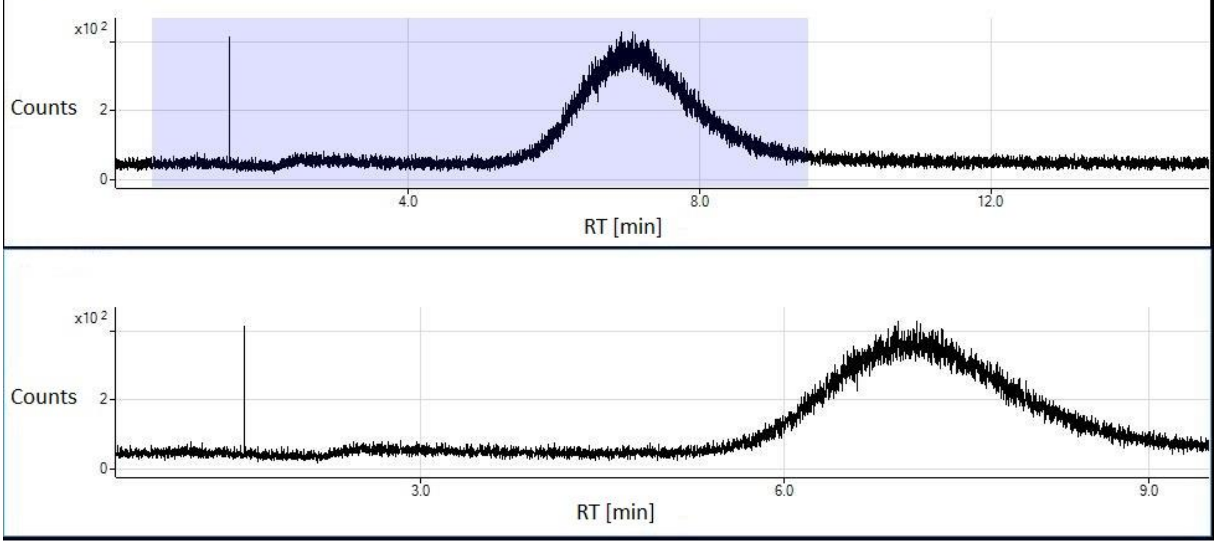
x10 2
Counts
0
2
4.0
8.0
12.0
RT [min]
x10 2
Counts
0
2
3.0
6.0
9.0
RT [min]

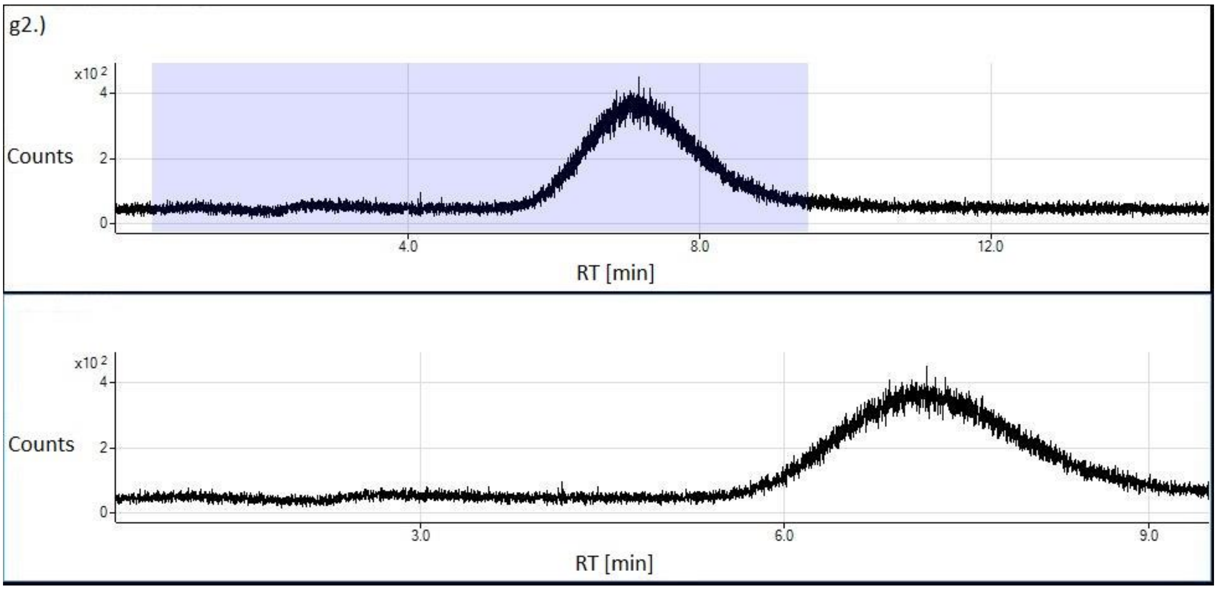
g2.)
x10 2
Counts
4
2
0
4.0
8.0
12.0
RT [min]
x10 2
Counts
4
2
0
3.0
6.0
9.0
RT [min]

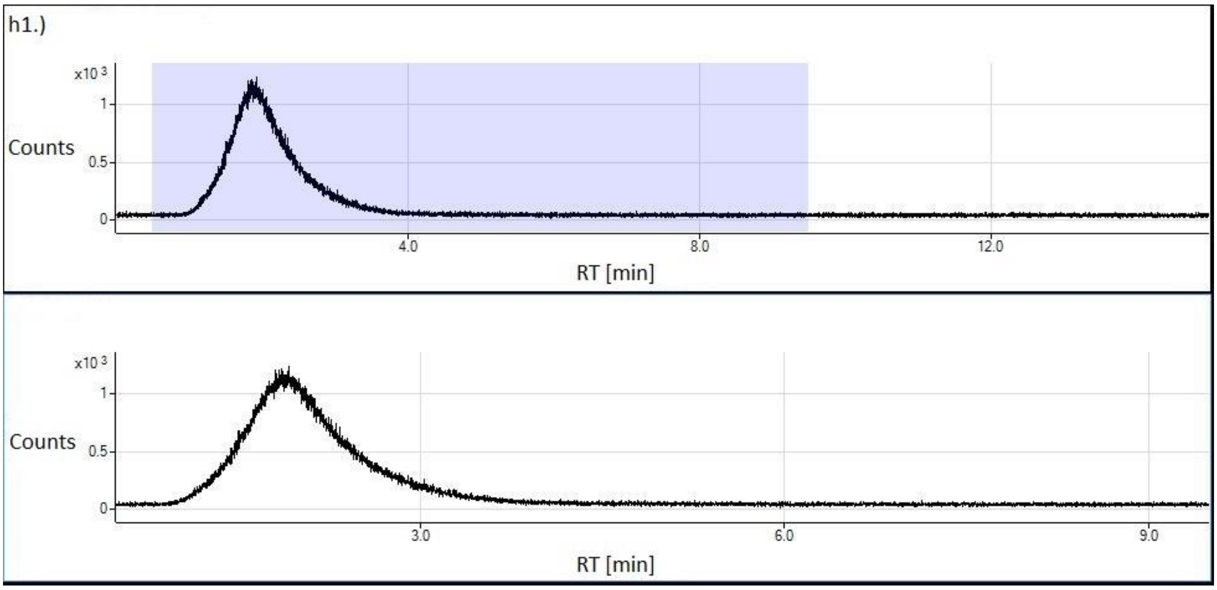
h1.)
x10 3
Counts
1
0.5
0
4.0
8.0
12.0
RT [min]
x10 3
Counts
1
0.5
0
3.0
6.0
9.0
RT [min]

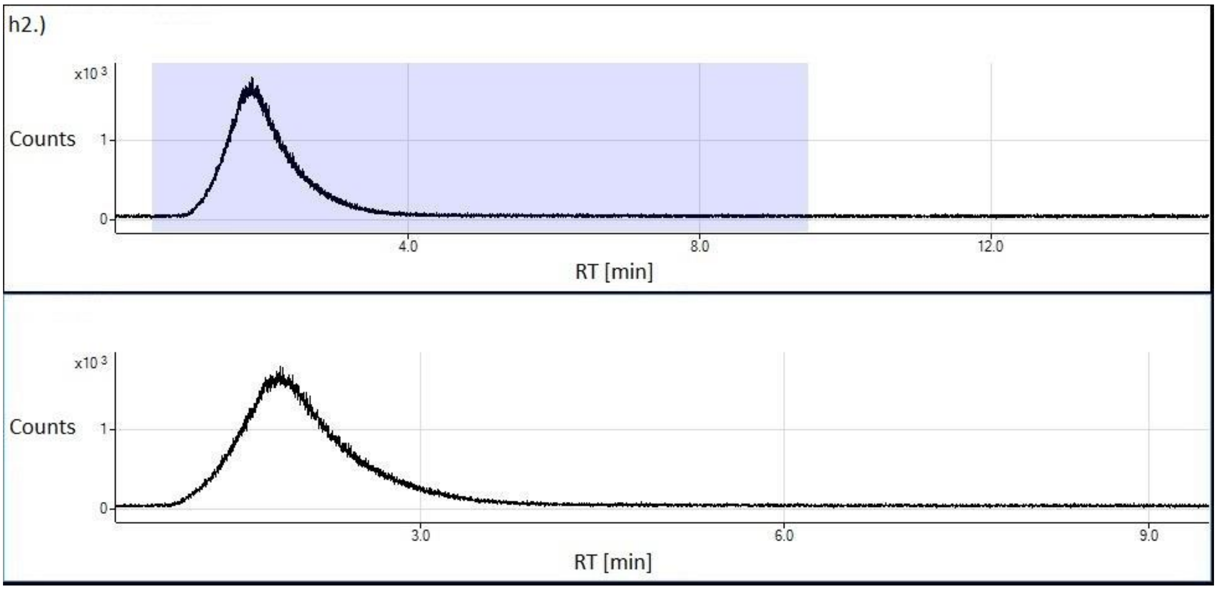
h2.)
x10 3
Counts
1
0
4.0
8.0
12.0
RT [min]
x10 3
Counts
1
0
3.0
6.0
9.0
RT [min]

Supplementary-Figure 7: Chromatograms from Cr redox speciation analysis by LC-ICP-MS for selected samples; Cr(III) peaks had a retention time (RT) below 4 minutes, Cr(VI) peaks had a RT above 5 minutes. Panel a, b, c and d: 50, 25, 12.5 and 2.5 ppb Cr(III) and Cr(VI) standards, respectively; Panel e1 and e2: Cr leached from 1 g L^{-1} chrysotile at pH 7.4 incubated for 168 h at a NaCl background of 250 mmol L^{-1} and an initial H_2O_2 concentration of 3.3 g L^{-1} (≈100 mmol L^{-1}) (sample is 10-fold diluted); f1 and f2: Cr leached from 1 g L^{-1} chrysotile at pH 7.4 incubated for 168 h at a NaCl background of 25 mmol L^{-1} and an initial H_2O_2 concentration of 3.3 g L^{-1} (≈100 mmol L^{-1}) (sample is 10-fold diluted); g1 and g2: Cr leached from 1 g L^{-1} chrysotile at unbuffered pH incubated for 168 h at a NaCl background of 250 mmol L^{-1} and an initial H_2O_2 concentration of 3.3 g L^{-1} (≈100 mmol L^{-1}) (sample is 5-fold diluted, pH at sampling was 8.8 with standard deviation of 0.1); h1 and h2: Cr leached from 1 g L^{-1} chrysotile at pH 3.0 incubated for 168 h at a NaCl background of 275 mmol L^{-1} (sample is 20-fold diluted). The chromatograms presented in the main text Figure 3 are the ones represented in panel a, e1 and i2. The Cr(III) and Cr(VI) concentrations calculated from the LC-ICP-MS data can be found in Supplementary Table 4.

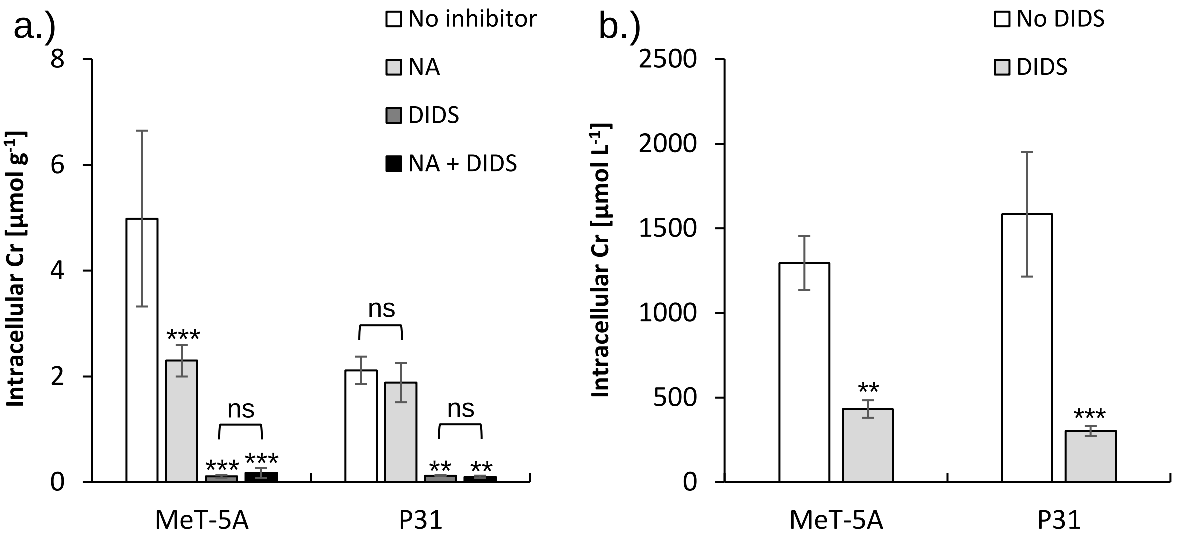

Supplementary-Figure 8: Panel a - Effect of 200 µmol L^{-1} of the anion transporter inhibitors DIDS and NA (and combinatory treatments with 200 µmol L^{-1} DIDS and 200 µmol L^{-1} NA) on measured intracellular Cr contents when 2 µmol L^{-1} of Cr(VI) were spiked to the media of mesothelial (MeT-5a) or mesothelioma (P31) cell cultures. Intracellular Cr contents in Panel a were normalized to the cellular protein contents. ** $p < 0.005$, *** $p < 0.001$ inhibitor versus no inhibitor, one-way ANOVA with Sidak´s multiple comparison test (ns indicates no statistically significant difference). Panel b - Effect of 200 µmol L^{-1} of the anion transporter inhibitor DIDS on intracellular Cr contents when 2000 µmol L^{-1} of Cr(VI) were spiked to the media of mesothelial (Met5a) or mesothelioma (P31) cell cultures. Intracellular Cr contents in Panel b were not normalized to the cellular protein contents in order to allow comparisons between the added concentration of DIDS and the extent of its inhibition of cellular Cr(VI) uptake. ** $p < 0.005$, *** $p < 0.001$ DIDS versus no DIDS, one-way ANOVA with Sidak´s multiple comparison test. Error bars indicate standard deviations (n = 3). Data to this Figure are presented in Supplementary-Table 17.

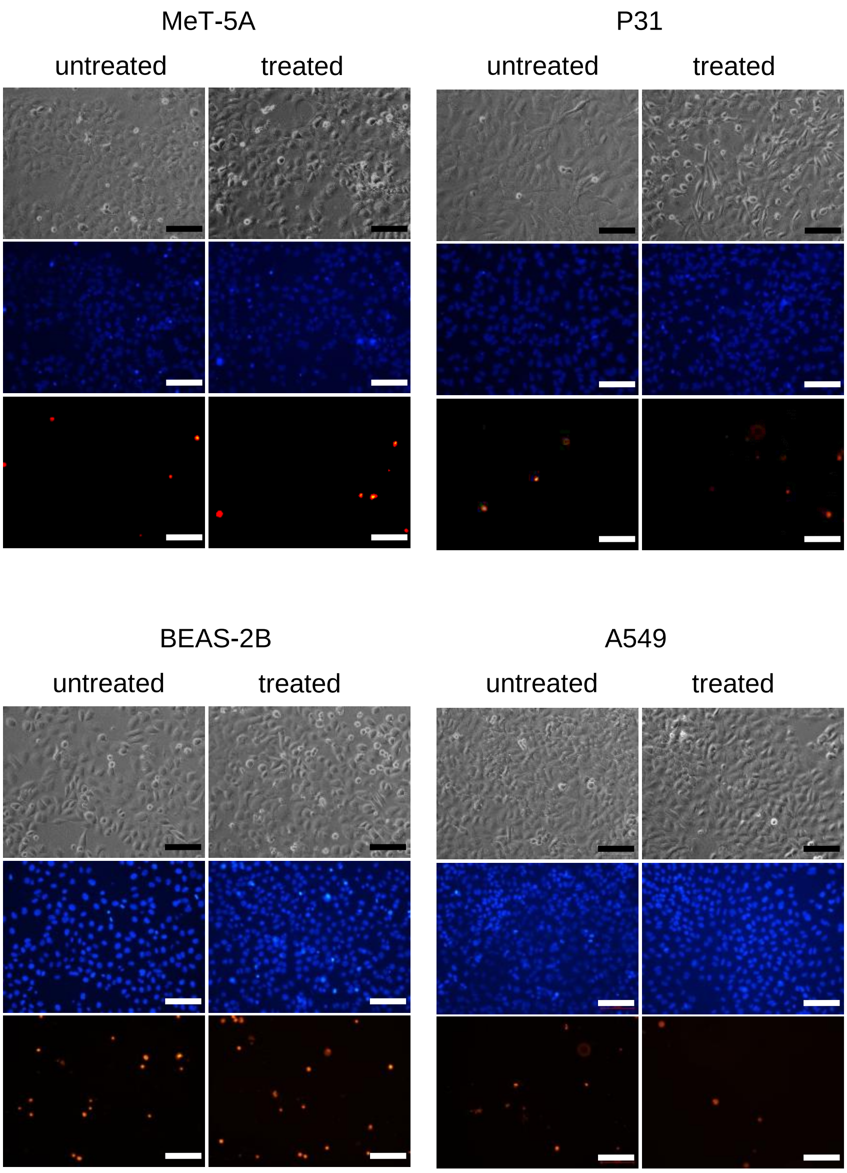

Supplementary-Figure 9: Absence of increased cytotoxicity in the Cr(VI) uptake experiment. MeT-5A, P31, BEAS-2B and A549 cells were seeded as for the cellular uptake experiment and treated with 2000 µmol L^{-1} Cr(VI) and 200 µmol L^{-1} each of the anion transporter inhibitors DIDS and NA. The fluorescent dyes Hoechst 33258 (blue) and propidium iodide (red) were added 1 h before micrographs were taken on a fluorescence microscope. Propidium iodide is taken up only by dead cells and thus indicates cytotoxicity. No increase in cytotoxicity was seen in any of the treated cell lines compared to their untreated counterparts after the 5 h treatment period. Scale bar = 100 µm.

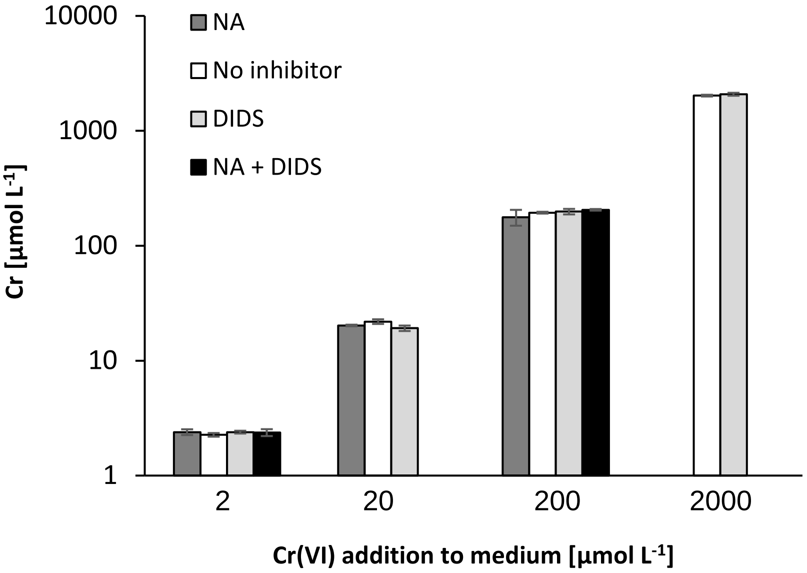

Supplementary-Figure 10: Panel a – Potential interference of 200 µmol L^{-1} of the anion transporter inhibitors DIDS and NA (and a combination of DIDS and NA) with the spiked Cr(VI) concentration of 2, 20, 200 and 2000 µmol L^{-1} in the cellular media used in the cellular uptake experiments. “No inhibitor” replicates are Cr(VI) spiked media samples without any inhibitor added. Error bars indicate standard deviations (n = 3). Data to this Figure are presented in Supplementary Table 18.

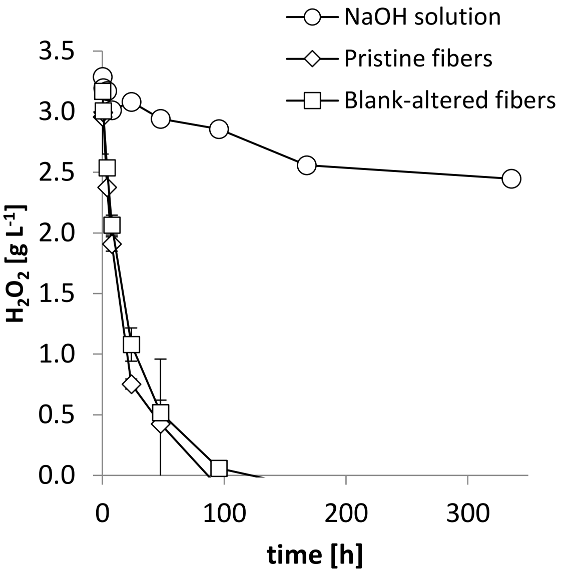

Supplementary-Figure 11: H_2O_2 degradation by 1 g L^{-1} pristine fibers and blank altered fibers in a 0.1 mol L^{-1} NaOH solution as a function of time (as reference, degradation in the NaOH buffer solution is also presented). Error bars indicate standard deviations (n = 2). Data to this Figure are presented in Supplementary Table 19.

Supplementary-Table 1: List of used cell lines, their type and source. Cell lines marked with an asterisk (*) were used for qPCR and cells marked with # were used for expression arrays. Cell lines marked with • were used in the Cr(VI) cell incubation experiments. Bronchial/Tracheal Epithelial Cell Growth Medium (BEG), Roswell Park Memorial Institute Medium 1640 (RPMI), Dulbecco´s Modified Eagle´s Medium (DMEM) and Minimal Essential Medium Eagle (MEM) were from Sigma.

Cell Line	Type	Medium	Source
BEAS-2B*•	immortalized lung epithelial	BEG	ATCC, Rockville
A549*•	lung carcinoma, epithelial	RPMI	ATCC, Rockville
MeT-5A*#•	immortalized mesothelial	RPMI	ATCC, Rockville
NP1*	primary mesothelial	RPMI	Medical University of Vienna
NP2*#	primary mesothelial	RPMI	Medical University of Vienna
I-2#	mesothelioma	RPMI	Ospedale Maggiore, Policlinico, Milano
M38K#	mesothelioma	RPMI	University of Helsinki
Meso-49#	mesothelioma	DMEM	Medical University of Vienna
Meso-62#	mesothelioma	DMEM	Medical University of Vienna
Meso-71#	mesothelioma	RPMI	Medical University of Vienna
Meso-80#	mesothelioma	RPMI	Medical University of Vienna
Meso-84#	mesothelioma	DMEM	Medical University of Vienna
Meso-92#	mesothelioma	DMEM	Medical University of Vienna
Meso-100#	mesothelioma	RPMI	Medical University of Vienna
Meso-103#	mesothelioma	DMEM	Medical University of Vienna
Meso-110#	mesothelioma	RPMI	Medical University of Vienna
Meso-189#	mesothelioma	RPMI	Medical University of Vienna
Meso-194#	mesothelioma	RPMI	Medical University of Vienna
Meso-200#	mesothelioma	RPMI	Medical University of Vienna
Meso-204#	mesothelioma	RPMI	Medical University of Vienna
Meso-205#	mesothelioma	RPMI	Medical University of Vienna

Meso-208#	mesothelioma	RPMI	Medical University of Vienna
Meso-221#	mesothelioma	RPMI	Medical University of Vienna
MM05*	mesothelioma	RPMI	The Prince Charles Hospital, Brisbane
P31*#•	mesothelioma	MEM	Umeå University
SPC111#	mesothelioma	RPMI	University of Zurich
SPC212*#	mesothelioma	RPMI	University of Zurich
VMC6#	mesothelioma	RPMI	Medical University of Vienna
VMC11#	mesothelioma	RPMI	Medical University of Vienna
VMC12#	mesothelioma	RPMI	Medical University of Vienna
VMC14#	mesothelioma	RPMI	Medical University of Vienna
VMC20#	mesothelioma	RPMI	Medical University of Vienna
VMC23*#	mesothelioma	RPMI	Medical University of Vienna
VMC28#	mesothelioma	RPMI	Medical University of Vienna
VMC31#	mesothelioma	RPMI	Medical University of Vienna
VMC33#	mesothelioma	RPMI	Medical University of Vienna
VMC40*#	mesothelioma	RPMI	Medical University of Vienna
VMC45#	mesothelioma	RPMI	Medical University of Vienna
VMC46#	mesothelioma	RPMI	Medical University of Vienna
VMC48#	mesothelioma	RPMI	Medical University of Vienna
VMC58#	mesothelioma	RPMI	Medical University of Vienna

Supplementary-Table 2: Data from Figure 1: Mobilized Cr concentrations from pristine fibers in the presence of 1 mmol L^{-1} of the metal chelators DTPA, EDTA, DFOB and in the absence of ligands (Blank). conc. indicates the measured Cr concentrations in solution, SD standard deviation.

Time	DTPA		EDTA		DFOB		Blank	
	conc.	SD	conc.	SD	conc.	SD	conc.	SD
[h]	[µmol L^{-1}]		[µmol L^{-1}]		[µmol L^{-1}]		[µmol L^{-1}]	
0.5	0.2	0.1	0.2	0.0	0.0	0.1	0.0	0.0
1	0.3	0.0	0.3	0.0	0.0	0.1	0.0	0.1
4	0.6	0.0	0.8	0.1	0.0	0.0	0.0	0.0
8	0.7	0.1	0.9	0.1	0.0	0.0	0.0	0.1
24	0.9	0.1	1.0	0.1	0.1	0.1	0.0	0.2
48	1.1	0.1	1.2	0.0	0.2	0.0	0.0	0.1
96	1.4	0.1	1.4	0.2	0.4	0.1	0.0	0.0
168	1.7	0.1	2.0	0.0	0.4	0.1	0.0	0.1
336	2.1	0.1	2.2	0.3	0.7	0.0	0.0	0.0

Supplementary-Table 3: Data from Figure 2: Mobilized Cr concentrations from pristine, blank-altered and DFOB-altered fibers in blank solutions and in the presence of 3.3 g L^{-1} H_2O_2 (starting concentration). conc. indicates the measured Cr concentrations in solution, SD standard deviations.

Panel a: Mobilized Cr concentrations from pristine fibers:

Time	Pristine fibers		Pristine fibers + H_2O_2	
	conc.	SD	conc.	SD
[h]	[µmol L^{-1}]		[µmol L^{-1}]	
0.5	0.0	0.0	0.2	0.0
1	0.0	0.1	0.4	0.0
4	0.0	0.0	0.6	0.2
8	0.0	0.1	0.8	0.1
24	0.0	0.2	1.1	0.1
48	0.0	0.1	1.4	0.1
96	0.0	0.0	1.7	0.0
168	0.0	0.1	2.1	0.1
336	0.0	0.0	2.3	0.0

Panel b: Mobilized Cr concentrations from blank-altered fibers:

Time	Blank-altered fibers		Blank-altered fibers + H_2O_2	
	conc.	SD	conc.	SD
[h]	[µmol L^{-1}]		[µmol L^{-1}]	
0.5	0.0	0.0	0.2	0.1
1	0.0	0.0	0.5	0.0
4	0.0	0.0	0.9	0.0
8	0.0	0.0	0.9	0.1
24	0.0	0.1	1.2	0.2
48	0.0	0.0	1.5	0.0
96	0.0	0.0	1.8	0.1
168	no data	no data	1.8	0.0
336	0.0	0.0	2.1	0.1

Panel c: Mobilized Cr concentrations from DFOB-altered fibers:

Time	DFOB-altered fibers		DFOB-altered fibers + H_2O_2	
	conc.	SD	conc.	SD
[h]	[µmol L^{-1}]		[µmol L^{-1}]	
0.5	0.1	0.2	0.2	0.2
1	0.2	0.1	0.3	0.0
4	0.0	0.0	0.4	0.2
8	0.0	0.1	0.6	0.1
24	0.2	0.0	0.9	0.1
48	0.2	0.1	1.2	0.0
96	0.2	0.2	1.4	0.2
168	no data	no data	1.9	0.0
336	0.2	0.1	2.0	0.1

Supplementary-Table 4: Data from Figure 3, additional Cr speciation data, and Cr mobilization data from treatments in the absence of the organic MOPS and PIPPS buffer. Mobilized Cr (total Cr and for selected samples Cr(III) and Cr(VI)), Mg and Si concentrations from pristine chrysotile fibers were measured for incubations under different conditions (pH, NaCl background concentration, fiber to solution ratio, applied H_2O_2 concentration, buffer medium). All samples were incubated for 168 h in an orbital shaker at 20 RPM. Cr(VI) was exclusively observed in samples to which H_2O_2 had been added. At pH 3.0, exclusively Cr(III) dissolved from fibers, indicating proton-promoted dissolution of bulk-Cr(III) from chrysotile. At neutral or mildly alkaline pH, the low Mg and Si dissolution indicate no dissolution beyond the first Mg layer, whereas at pH 3.0 the increased Mg and Si dissolution indicates dissolution beyond this first layer (Walter et al. 2019, reference No. 15 in the main text). Chromatograms of the LC-ICP-MS analyses for determining the Cr redox speciation in this table are presented in Supplementary-Figure 7. Values in round braces are standard deviations (n = 2). N/A means not applicable, b.d. means below detection, b.q. means below quantification.

**Targeted pH**	**pH measured at sampling**	**NaCl background**	**Fiber to solution ratio**	**H_2O_2 concentrations at start**	**Buffer / Medium**	**Total Cr**	**Cr(III)**	**Cr(VI)**	**Mg**	**Si**
		[mmol L^{-1}]	[g L^{-1}]	[mmol L^{-1}]		[µmol L^{-1}] *ICP-MS*	[µmol L^{-1}] *LC-ICP-MS*	[µmol L^{-1}] *LC-ICP-MS*	[µmol L^{-1}] *ICP-OES*	[µmol L^{-1}] *ICP-OES*
7.4 [a, b]	6.9 (0.0)	250	1	100	MOPS	2.08 (0.21)	b.d.	2.04 (0.01)	613 (49.7)	b.q.
7.4	7.5 (0.0)	25	1	0	MOPS	0.01 (0.00)	N/A	N/A	584 (14.3)	21.6 (0.0)
7.4	7.0 (0.1)	25	1	100	MOPS	1.75 (0.07)	b.d.	1.82 (0.08)	753 (303)	33.9 (1.9)
N/A	8.9 (0.0)	250	1	0	N/A	0.04 (0.01)	N/A	N/A	131 (0.1)	b.q.
N/A	8.8 (0.1)	250	1	100	N/A	0.69 (0.02)	b.d.	0.64 (0.01)	156 (1.3)	b.q.
3.0 [c]	3.3 (0.0)	275	1	0	PIPPS	5.13 (1.53)	5.60 (1.33)	b.d.	1690 (516)	355 (78.4)

[a] As no Cr had been detected in the experiments of Figure 2a (main text), no sample without H_2O_2 was included for this treatment in the speciation analyses.
[b] For the same treatment at a fiber to solution ratio of 25 g L^{-1}, LC-ICP-MS analyses also exclusively detected Cr(VI) (data not shown).
[c] No notable differences (including Cr speciation) were observed in the same treatment at 10-fold decreased NaCl background (data not shown).

Supplementary-Table 5: Removal of Mg, Si, Fe and Ni from fibers at pH 7.4 during preconditioning with 1 mmol L^{-1} ligand or blank solutions for 336 h (first column), and during interaction of pristine and preconditioned fibers with a 3.3 g L^{-1} H_2O_2 solution (second column) and a 1 mmol L^{-1} DFOB solution (third column) for 336 h. Values in round braces indicate standard deviations (n = 2). Standard deviations could not be determined for fiber preconditioning carried out in a single container.

	**Removal during preconditioning with or without ligands**	**Removal from (preconditioned) fibers by 3.3 g L^{-1} H_2O_2**	**Removal from preconditioned fibers by 1 mmol L^{-1} DFOB**
	[µmol L^{-1}]	[µmol L^{-1}]	[µmol L^{-1}]
**Mg**			
Pristine fibers	-	602 (19)	-
DFOB-altered fibers	604	229 (13)	248 (2)
DTPA-altered fibers	829 (55)	180 (13)	-
EDTA-altered fibers	974 (24)	189 (14)	-
Blank-altered fibers	530	91 (7)	-
**Si**			
Pristine fibers	-	23 (1)	-
DFOB-altered fibers	81	117 (10)	171 (1)
DTPA-altered fibers	211 (14)	113 (8)	-
EDTA-altered fibers	263 (19)	119 (12)	-
Blank-altered fibers	13	35 (1)	-
**Fe**			
Pristine fibers	-	0.1 (0.1)	-
DFOB-altered fibers	29 (0.2)	0.7 (0.3)	4.2 (0.4)
DTPA-altered fibers	36 (3.9)	1.1 (0.1)	-
EDTA-altered fibers	40 (2.6)	0.0 (0.1)	-
Blank-altered fibers	0	0.2 (0.3)	-
**Ni**			
Pristine fibers	-	0.8 (0.2)	-
DFOB-altered fibers	1.7 (0.0)	0.4 (0.1)	0.3 (0.0)
DTPA-altered fibers	1.9 (0.2)	0.4 (0.2)	-
EDTA-altered fibers	2.0 (0.1)	0.2 (0.2)	-
Blank-altered fibers	0.2	0.6 (0.1)	-

Supplementary-Table 6: Cr speciation analysis: The valence of the dissolved Cr in the pH 7.4 H_2O_2 leaching experiments was – apart from the LC-ICP-MS method – additionally assessed by equilibrium modelling. The solution speciation was predicted for an experimental sample using the geochemical modelling program PHREEQC and the SIT database (reference No. 82 in the main text). The total dissolved element concentrations (leached elements from the asbestos fibers and the NaCl added as background electrolyte) in the sample with the largest leached Cr concentration (2.3 µmol L^{-1} Cr; pristine fibers; t = 336 h) were used as model input. Additionally, the pH and pe of the models were fixed to 7.4 and 12.5, respectively. At pH 7.4, the pe value of 12.5 reflects a solution in equilibrium with the atmospheric oxygen partial pressure, which is representative for the experimental conditions.

Predicted solution speciation of the Cr leached by H_2O_2 after 336 h at pH 7.4:

**Solution speciation modelling**<br>Input: 2.3 µmol L^{-1} Cr (no pre-specified valence)		
Most abundant species of the different Cr valences		Molarity [µmol L^{-1}]
Cr(II)	$Cr(OH)^+$ & Cr^{2+}	0.00e+00
Cr(III)	$Cr(OH)_3$	4.23e-15
	$Cr(OH)_2^+$	9.23e-17
Cr(VI)	CrO_4^{2-}	2.20e-00
	$HCrO_4^-$	1.02e-01

Electrolyte and measured element concentrations used as input data for the solution speciation modelling:

**Element**	**Input concentration [µmol L^{-1}]**	**Element**	**Input concentration [µmol L^{-1}]**
Na	250000	Cr	2.3
Cl	250000	Zn	0.8
Mg	601.8	Al	0.7
Si	23.0	Ni	0.3
Mn	4.5	Fe	0.1

Supplementary-Table 7: Data from Figure 4. Expression of Cr(VI) transporter proteins, and uptake of Cr(VI), in cells of typically asbestos burdened tissues. conc. indicates the measured Cr concentrations in solution, SD standard deviations.

Panel a.) Expression of SLC4A1 and SLC26A1 in immortalized mesothelial, mesothelioma, immortalized lung epithelial and lung carcinoma cells assessed by qPCR. Beta-actin and GAPDH were used as housekeeping genes. Reported values are the means of SLC4A1 or SLC26A1 as detected relative to Beta-actin and GAPDH:

Tissue type	Cell line	Transporter gene	
Mesothelium		SLC4A1	SLC26A1
	NP1	0.8	201
	NP2	1.7	256
	MeT-5A	34.9	164
Mesothelioma		SLC4A1	SLC26A1
	VMC40	1.1	145
	MM05	14.6	76.3
	SPC212	1.0	23.0
	P31	14.0	118
	VMC23	0.3	62.2
Lung		SLC4A1	SLC26A1
	BEAS-2B	2.3	129
Lung carcinoma			
		SLC4A1	SLC26A1
	A549	785	171

Panel b.) Expression levels of SLC4A1 and SLC26A1 (two oligos, v1 and v1) in a panel of mesothelial (n=2) and mesothelioma (n=35) cell cultures were extracted from Agilent 44K microarray data and are shown as raw hybridization signal:

Mesothelioma cell lines			Normal mesothelium		
SLC26A1 v1	SLC26A1 v2	SLC4A1	SLC26A1 v1	SLC26A1 v2	SLC4A1
465	5	7	2279	76	92
4218	8	49	2114	125	190
271	38	16	-	-	-
210	42	8	-	-	-
523	114	7	-	-	-
2921	4	13	-	-	-
2532	11	11	-	-	-
3032	155	34	-	-	-
1840	58	11	-	-	-
299	52	12	-	-	-
111	116	10	-	-	-
72	40	7	-	-	-
144	55	5	-	-	-
385	28	19	-	-	-
116	27	9	-	-	-
809	35	16	-	-	-
874	96	12	-	-	-
341	30	20	-	-	-
833	6	15	-	-	-
4635	3	50	-	-	-
369	72	16	-	-	-
183	22	10	-	-	-
269	26	7	-	-	-
320	9	21	-	-	-
406	15	19	-	-	-
264	12	10	-	-	-
558	20	28	-	-	-
3900	33	33	-	-	-
3600	43	46	-	-	-
6341	68	61	-	-	-
3093	33	47	-	-	-
327	46	8	-	-	-
283	36	30	-	-	-
1496	59	29	-	-	-
2748	64	15	-	-	-

Panel d: Intracellular Cr(VI) levels (normalized to the cellular protein, in µmol g^{-1}) in MeT-5A, P31, BEAS-2B and A549 cells when either 0, 0.2, 2, 20, 200 and 200 µmol L^{-1} Cr(VI) were spiked into the cell incubation media:

Spike-in Cr(VI) concentration [µmol L^{-1}]	MeT-5A		P31		A549		BEAS-2B	
	conc. [µmol g^{-1}]	SD	conc. [µmol g^{-1}]	SD	conc. [µmol g^{-1}]	SD	conc. [µmol g^{-1}]	SD
0	0.06	0.04	0.02	0.01	0.04	0.06	0.05	0.02
0.2	0.89	0.69	0.36	0.07	0.29	0.03	0.35	0.12
2	5.0	1.7	2.1	0.26	1.9	0.26	2.2	0.39
20	26.6	3.4	25.5	1.7	15.2	3.3	16.0	1.7
200	115	59.4	91.6	4.0	80.6	25.5	79.2	11.4
2000	286	111	209	12.5	260	42.2	202	35.2

Panel e: Intracellular Cr(VI) levels (normalized to the cellular protein, in µmol g^{-1}) in MeT-5A, P31, BEAS-2B and A549 cells when the cell media were spiked with 2 µmol L^{-1} of Cr(VI)) with or without the presence of 200 µmol L^{-1} of the anion transporter inhibitor DIDS:

	MeT-5A		P31		BEAS-2B		A549	
	conc. [µmol g^{-1}]	SD	conc. [µmol g^{-1}]	SD	conc. [µmol g^{-1}]	SD	conc. [µmol g^{-1}]	SD
No DIDS	5.0	1.7	2.1	0.26	2.2	0.39	1.9	0.26
DIDS	0.11	0.03	0.12	0.01	0.15	0.04	0.26	0.10

Panel f: Intracellular Cr(VI) (not normalized to the cellular protein content, in µmol g^{-1}) in MeT-5A, P31, BEAS-2B and A549 cells as compared to measured Cr(VI) concentrations in the cell media alone ("Medium" replicates) when 0.2, 2, 20, 200 and 2000 µmol L^{-1} Cr(VI) were spiked into the cell media:

Spike-in Cr(VI) concentration [µmol L^{-1}]	Medium		MeT-5A		P31		A549		BEAS-2B	
	conc. [µmol L^{-1}]	SD	conc. [µmol L^{-1}]	SD	conc. [µmol L^{-1}]	SD	conc. [µmol L^{-1}]	SD	conc. [µmol L^{-1}]	SD
0	0.36	0.33	0.45	0.27	0.15	0.06	0.41	0.62	0.23	0.06
0.2	0.45	0.03	3.2	0.57	3.1	0.85	2.7	0.16	1.2	0.27
2	2.3	0.08	21.0	1.6	19.4	12.3	17.0	2.6	12.5	2.0
20	21.9	1.0	169	34.8	216	11.1	137	43.3	79.8	35.2
200	194	3.4	546	147	718	116	642	221	484	28.7
2000	2024	34.2	1294	159	1583	368	2115	121	1272	50.0

Supplementary-Table 8: Data from Figure 5. H_2O_2 decomposition by pristine and differently preconditioned chrysotile fibers, and by MOPS buffer alone, and hydroxyl radical yield of differently preconditioned chrysotile fibers.

Panel a.) H_2O_2 concentrations in 1 g L^{-1} preconditioned fiber suspensions or MOPS buffer. The starting concentration of H_2O_2 was 3.3 g L^{-1}:

Time [h]	MOPS buffer (DTPA & EDTA) [g L^{-1}]	SD	DTPA-altered fibers [g L^{-1}]	SD	Pristine fibers [g L^{-1}]	SD	EDTA-altered fibers [g L^{-1}]	SD	MOPS buffer (DFOB) [g L^{-1}]	SD	DFOB-altered fibers [g L^{-1}]	SD
96	2.71	0.04	2.66	0.01	1.84	0.05	2.60	0.03	3.0	0.04	2.50	0.02
168	2.61	0.03	2.51	0.04	1.20	0.02	2.52	0.00	2.8	0.03	2.00	0.09
336	2.32	0.02	2.19	0.06	0.40	0.26	2.21	0.05	2.6	0.02	1.50	0.02

SD: standard deviation (n = 2).

Panel b.) HO• yield of EDTA-altered, DTPA-altered and DFOB-altered fibers relative to pristine fibers (HO• yield of pristine fibers = 100%):

Blank-altered fibers [%]	SD	EDTA-altered fibers [%]	SD	DTPA-altered fibers [%]	SD	DFOB-altered fibers [%]	SD
61.5	10.9	13.8	3.5	3.2	0.9	8.8	2.5

SD: standard deviation (n = 4).

Supplementary-Table 9: Efficacy of the HNO_3 extraction (5 g L^{-1} pristine chrysotile extracted in 1.4 mol L^{-1} HNO_3 at 50°C for 2 weeks) of pristine Shijiazhuang chrysotile to dissolve the Mg content of the fibers. Values in round braces indicate standard deviations (n = 3).

**Mean Mg concentration extracted in HNO_3 digestion of pristine chrysotile fibers:** [g kg^{-1}]	**Bulk Mg concentrations of pristine fibers as determined in fusion digestions (Table 1 in main text):** [g kg^{-1}]	**Efficiency of the HNO_3 digest (HNO_3 digestion/fusion digestion bulk * 100%)** [%]
240 (1.9)	253 (8.9)	95

Supplementary-Table 10: Predicted solution speciation of the Cr leached from blank-altered fibers by H_2O_2 in the leaching experiment of Supplementary-Figure 1b after 336 h (sample with the largest Cr concentration) using PhreeqC and the SIT database. This modeling was carried out similarly as for the results presented in Supplementary-Table 6. For this prediction, however, pH 13 and pe 6 were used as input data. The results of this modelling calculation are presented in the table below:

**Solution speciation modelling**<br>Input: 1.3 µmol L^{-1} Cr (no pre-specified valence)		
Most abundant species of the different Cr valences		Molarity [µmol L^{-1}]
Cr(II)	$Cr(OH)^+$ & Cr^{2+}	0.00e+00
Cr(III)	$Cr(OH)_4^-$	3.07e-22
	$Cr(OH)_3$	1.13e-23
Cr(VI)	CrO_4^{2-}	1.30e-00
	$HCrO_4^-$	1.99e-07

The elemental model input data (the leached elemental concentrations from the asbestos fibers and the added NaCl background) are presented in the table below:

**Element**	**Input concentration [µmol L^{-1}]**	**Element**	**Input concentration [µmol L^{-1}]**
Na	100000	Zn	0.4
Mg	3.3	Al	6.3
Si	16.5	Ni	0.3
Mn	0.03	Fe	0.4
Cr	1.3		

The results of this modeling calculation suggest that the leached Cr from blank-altered fibers in the experiment of Supplementary-Figure 1b was Cr(VI).

Supplementary-Table 11: Data underlying Supplementary-Figure 1: Analyses and experiments to test whether mineral impurities in the Shijiazhuang chrysotile material could have significantly contributed to the observed Cr leaching from chrysotile fibers. conc. indicates the measured Cr concentrations in solution, SD indicates standard deviations (Panel a, b and d: n = 2, Panel c: n = 3), N/A means not applicable.

Panel a: Extraction efficacy of Fe and Cr in a HNO_3 fiber extraction (5 g L^{-1} pristine chrysotile digested in 1.4 mol L^{-1} HNO_3 at 50°C for 2 weeks) in relation to the Cr and Fe bulk in Shijiazhuang chrysotile as determined by fusion digestions and/or a neutron activation analysis (NAA):

	Fe (in NAA)		Fe (rel. to NAA)		Cr (rel. to fusion dig.)		Cr (rel. to NAA)	
	conc. [µmol L^{-1}]	SD	conc. [µmol L^{-1}]	SD	conc. [µmol L^{-1}]	SD	conc. [µmol L^{-1}]	SD
Element dissolved in HNO_3 digest	N/A	N/A	66.8	3.0	100.0	7.9	92.9	2.9
Fe in magnetite	31.8	N/A	N/A	N/A	N/A	N/A	N/A	N/A
Fe in chrysotile	68.2	N/A	N/A	N/A	N/A	N/A	N/A	N/A

Panel b: Cr leaching from pristine and blank-altered chrysotile fibers (1 g L^{-1}) by 3.3 g L^{-1} H_2O_2 (starting concentration) under alkaline conditions (0.1 mol L^{-1} NaOH) at which chrysotile does not dissolve:

Time	Pristine fibers + H_2O_2		Blank altered fibers + H_2O_2		Pristine fibers no H_2O_2	
[h]	conc. [µmol L^{-1}]	SD	conc. [µmol L^{-1}]	SD	conc. [µmol L^{-1}]	SD
0.5	0.1	0.1	0.4	0.0	N/A	N/A
1	0.1	0.1	0.6	0.1	N/A	N/A
4	0.1	0.1	0.6	0.0	N/A	N/A
8	0.1	0.1	1.0	0.2	N/A	N/A
24	0.3	0.1	1.1	0.0	N/A	N/A
48	0.2	0.1	1.2	0.1	N/A	N/A
96	0.2	0.1	1.3	0.0	N/A	N/A
168	0.3	0.0	1.3	0.2	N/A	N/A
336	0.4	0.1	1.3	0.0	0.1	0.1

Panel c: Cumulatively mobilized Cr concentrations in different fiber treatments correlated to cumulatively mobilized Mg:

	Cr		Mg	
	conc.	SD	conc.	SD
	[µmol g^{-1}]		[µmol g^{-1}]	
DFOB & H_2O_2 incubation	2.7	0.00	833	18.9
DTPA & H_2O_2 incubation	3.6	0.10	1008	13.0
EDTA & H_2O_2 incubation	4.1	0.10	1163	13.5
Blank & H_2O_2 incubation	2.1	0.10	621	14.1
H_2O_2 incubation	2.3	0.09	602	6.6

Panel d: Cumulatively mobilized Cr concentrations in different fiber treatments correlated to cumulatively mobilized Si:

	Cr		Si	
	conc.	SD	conc.	SD
	[µmol g^{-1}]		[µmol g^{-1}]	
DFOB & H_2O_2 incubation	2.7	0.00	198	10.0
DTPA & H_2O_2 incubation	3.6	0.10	323	7.8
EDTA & H_2O_2 incubation	4.1	0.10	382	11.7
Blank & H_2O_2 incubation	2.1	0.10	48.3	1.3
H_2O_2 incubation	2.3	0.09	22.5	0.89

Supplementary-Table 12: Data to Supplementary-Figure 2. PCR amplification efficiencies of the primer pairs used in the qPCR measurements of the study:

log10 of template concentration	mean Ct value			
	SLC4A1	SLC26A1	GAPDH	β-actin
0	29.6	25.5	12.9	11.1
-0.301	31.5	26.3	14.9	12.8
-0.481	34.2	27.1	15.6	13.8
-0.602	32.2	27.2	15.5	13.9
-1	34.6	28.2	16.4	14.6
-2	36.8	32.4	20.0	18.0

Supplementary-Table 13: Data from Supplementary-Figure 3: Mobilized Ni and Fe concentrations from pristine fibers in the presence of 1 mmol L^{-1} of the metal chelators DTPA, EDTA, DFOB and in the absence of ligands (Blank). conc. indicates the measured Ni or Fe concentrations in solution, SD indicates standard deviations (n=2). The data for DFOB and blank Fe mobilization were taken from Walter et al. (2019) (Reference No. 15 in the main text).

Panel a: Mobilized Ni concentration:

Time	DTPA		EDTA		DFOB		Blank	
	conc.	SD	conc.	SD	conc.	SD	conc.	SD
[h]	[µmol L^{-1}]		[µmol L^{-1}]		[µmol L^{-1}]		[µmol L^{-1}]	
0.5	0.3	0.1	0.5	0.1	0.4	0.1	0.0	0.0
1	0.4	0.2	0.6	0.1	0.4	0.0	0.2	0.2
4	0.7	0.0	0.9	0.1	0.8	0.2	0.2	0.0
8	0.9	0.1	1.3	0.1	0.7	0.1	0.2	0.1
24	1.4	0.5	1.7	0.2	1.1	0.1	0.2	0.1
48	1.4	0.0	1.6	0.1	1.4	0.0	0.3	0.0
96	1.7	0.2	2.0	0.0	1.5	0.2	0.4	0.1
168	1.9	0.1	2.2	0.0	1.2	0.2	0.3	0.0
336	2.2	0.3	2.4	0.0	1.7	0.0	0.3	0.0

Panel b: Mobilized Fe concentrations:

Time	DTPA		EDTA		DFOB		Blank	
	conc.	SD	conc.	SD	conc.	SD	conc.	SD
[h]	[µmol L^{-1}]		[µmol L^{-1}]		[µmol L^{-1}]		[µmol L^{-1}]	
0.5	6.7	0.2	11.3	0.6	4.1	0.3	0.1	0.0
1	9.4	0.1	14.9	1.6	5.7	0.4	0.0	0.0
4	16.9	1.2	24.6	3.1	13.7	1.2	0.2	0.1
8	21.3	0.6	29.3	0.3	15.1	1.5	0.0	0.0
24	29.3	6.6	36.5	1.8	22.6	2.4	0.0	0.1
48	31.3	5.7	35.9	1.5	27.5	3.8	0.0	0.0
96	39.5	1.4	39.6	5.2	32.4	4.1	0.0	0.1
168	45.5	2.5	49.7	1.7	27.3	0.1	0.0	0.0
336	44.8	0.8	45.1	1.3	29.3	0.2	0.1	0.0

Supplementary-Table 14: Data from Supplementary-Figure 4: Mobilized Cr concentrations from pristine chrysotile in the presence of 0, 0.1, 1, 10 or 100 mmol L^{-1} H_2O_2 (initial concentrations). Fibers were incubated at pH 7.4 for 168 h in an orbital shaker at 20 RPM. This experiment was conducted at a 10 times lower NaCl background concentration (suprapur quality, Sigma) to facilitate ultra-trace metal analysis. St.Dev. means standard deviation.

H_2O_2 at the beginning of the experiment [mmol L^{-1}]		Mean of mobilized Cr (n=2)	St.Dev. of mobilized Cr (n=2)
0	[nmol L^{-1}]	13.2	2.9
0.1	[nmol L^{-1}]	19.2	0.5
1	[µmol L^{-1}]	0.37	0.01
10	[µmol L^{-1}]	0.93	0.01
100	[µmol L^{-1}]	1.75	0.07

Supplementary-Table 15: Data from Supplementary-Figure 5: Cr mobilization at pH 3.0 ± 0.3 (PIPPS) and 7.4 ± 0.3 (MOPS) from 1 g L^{-1} chrysotile suspensions. Incubation times ranged from 0.5 to 336 h. conc. indicates the measured Cr concentrations in solution, SD indicates standard deviations (n=2).

Time	pH 3.0		pH 7.4	
	conc.	SD	conc.	SD
[h]	[µmol L^{-1}]		[µmol L^{-1}]	
0.5	1.2	0.2	0.0	0.0
1	1.0	0.1	0.0	0.1
2	1.2	0.0	0.0	0.1
4	1.3	0.4	0.0	0.0
8	1.9	0.1	0.0	0.1
24	2.4	0.2	0.0	0.2
48	2.8	0.3	0.0	0.1
96	3.3	0.2	0.0	0.0
168	4.4	0.1	0.0	0.1
336	6.1	0.1	0.0	0.0

Supplementary-Table 16: Data from Supplementary-Figure 6: Mobilized Ni concentrations from pristine, blank-altered and DFOB-altered fibers in blank solutions and in the presence of 3.3 g L^{-1} H_2O_2 (starting concentration). conc. indicates the measured Cr concentrations in solution, SD indicates standard deviations (n=2). No data on Fe are presented as neither in blank nor in H_2O_2 samples concentrations exceeded the limit of qualification.

Panel a: Mobilized Ni concentrations from pristine fibers:

Time	Pristine fibers		Pristine fibers + H_2O_2	
	conc.	SD	conc.	SD
[h]	[µmol L^{-1}]		[µmol L^{-1}]	
0.5	0.0	0.0	0.4	0.1
1	0.2	0.2	0.3	0.1
4	0.2	0.0	0.4	0.1
8	0.2	0.1	0.5	0.1
24	0.2	0.1	0.4	0.0
48	0.3	0.0	0.4	0.3
96	0.4	0.1	0.6	0.0
168	0.3	0.0	0.6	0.1
336	0.3	0.0	0.8	0.2

Panel b: Mobilized Ni concentrations from blank-altered fibers:

Time	Blank-altered fibers		Blank-altered fibers + H_2O_2	
	conc.	SD	conc.	SD
[h]	[µmol L^{-1}]		[µmol L^{-1}]	
0.5	0.4	0.2	0.2	0.0
1	0.6	0.0	0.3	0.0
4	0.3	0.4	0.3	0.0
8	0.3	0.2	0.2	0.1
24	0.3	0.1	0.3	0.1
48	0.3	0.0	0.3	0.1
96	0.4	0.1	0.4	0.1
168	no data	no data	0.4	0.1
336	0.4	0.2	0.6	0.1

Panel c: Mobilized Ni concentrations from DFOB-altered fibers:

Time	DFOB-altered fibers		DFOB-altered fibers + H_2O_2	
	conc.	SD	conc.	SD
[h]	[µmol L^{-1}]		[µmol L^{-1}]	
0.5	0.1	0.1	0.2	0.1
1	0.0	0.0	0.2	0.0
4	0.2	0.0	0.2	0.1
8	0.3	0.2	0.4	0.0
24	0.0	0.2	0.3	0.1
48	0.1	0.0	0.4	0.0
96	0.2	0.1	0.4	0.1
168	no data	no data	0.2	0.1
336	0.3	0.1	0.4	0.1

Supplementary-Table 17: Data to Supplementary-Figure 8. Effects of inhibitors NA or DIDS on the measured intracellular Cr(VI) concentrations in mesothelial derived cells (MeT-5A: mesothelial; P31: mesothelioma). conc. indicates the measured cellular Cr concentrations, SD indicates standard deviations (n = 3).

Panel a: Intracellular Cr concentrations (normalized to the cellular protein content) when 200 µmol L^{-1} of the anion transporter inhibitors DIDS and NA (and combinatory treatments with 200 µmol L^{-1} DIDS and 200 µmol L^{-1} NA) and 2 µmol L^{-1} of Cr(VI) were spiked to the media of MeT-5a or P31 cell cultures.

	Met5a		P31	
	conc.	SD	conc.	SD
	[µmol g^{-1}]		[µmol g^{-1}]	
No inhibitor	5.0	1.7	2.1	0.26
NA	2.3	0.30	1.9	0.37
DIDS	0.11	0.03	0.12	0.01
NA + DIDS	0.17	0.09	0.10	0.02

Panel b: Intracellular Cr concentrations (not normalized to the cellular protein contents in order to allow comparisons between the added concentration of DIDS and the amount of its inhibition of cellular Cr(VI) uptake) when 200 µmol L^{-1} of the anion transporter inhibitor DIDS and 2000 µmol L^{-1} of Cr(VI) were spiked to the media of Met5a or P31 cell cultures.

	Met5a		P31	
	conc.	SD	conc.	SD
	[µmol L^{-1}]		[µmol L^{-1}]	
No DIDS	1294	159	1583	368
DIDS	432	51.5	303	29.6

Supplementary-Table 18: Data to Supplementary Figure 10: Cr concentrations measured in the medium when 200 µmol L^{-1} of the anion transporter inhibitors DIDS and NA (and a combination of DIDS and NA) and Cr(VI) concentration of 2, 20, 200 and 2000 µmol L^{-1} were added to the cellular media. "No inhibitor" replicates are Cr(VI) spiked media samples without any inhibitor added. conc. indicates the measured medium Cr concentrations, SD indicates standard deviations (n = 3).

	2 µmol L^{-1}		20 µmol L^{-1}		200 µmol L^{-1}		2000 µmol L^{-1}	
	conc.	SD	conc.	SD	conc.	SD	conc.	SD
	[µmol L^{-1}]		[µmol L^{-1}]		[µmol L^{-1}]		[µmol L^{-1}]	
No inhibitor	2.3	0.08	21.9	1.0	194	3.4	2024	34.2
DIDS	2.4	0.07	19.2	1.0	198	10.9	2081	60.5
NA	2.4	0.14	20.2	0.33	177	27.9	-	-
NA + DIDS	2.4	0.16	-	-	205	3.2	-	-

Supplementary-Table 19: Data to Supplementary Figure 11. H_2O_2 degradation by 1 g L^{-1} pristine fibers and blank-altered fibers in a 0.1 mol L^{-1} NaOH solution as a function of time (as reference, degradation in the NaOH buffer solution is also presented). conc. indicates the measured H_2O_2 concentrations in solution, SD indicates standard deviations (n = 2).

Time	NaOH solution		Pristine fibers		Blank-altered fibers	
	conc.	SD	conc.	SD	conc.	SD
[h]	[g L^{-1}]		[g L^{-1}]		[g L^{-1}]	
0.5	3.29	0.01	2.96	0.31	3.17	0.02
1	3.20	0.05	2.99	0.00	3.01	0.02
4	3.17	0.05	2.38	0.02	2.54	0.03
8	3.01	0.04	1.91	0.06	2.06	0.08
24	3.08	0.04	0.75	0.04	1.08	0.14
48	2.94	0.01	0.43	0.53	0.52	0.10
96	2.86	0.03	0.00	0.00	0.06	0.03
168	2.56	0.03	0.00	0.00	0.00	0.00
336	2.45	0.00	0.00	0.00	0.00	0.00
